# Trimetallic FeCoNi Metal–Organic Framework with Enhanced Peroxidase-like Activity for the Construction of a Colorimetric Sensor for Rapid Detection of Thiophenol in Water Samples

**DOI:** 10.3390/molecules29163739

**Published:** 2024-08-07

**Authors:** Zehui Deng, Jiaqing Cao, Lei Zhao, Zhao Zhang, Jianwei Yuan

**Affiliations:** 1 State Key Laboratory of Pollution Control and Resource Reuse, School of Environment, Nanjing University, Nanjing 210023, China; dg1925004@smail.nju.edu.cn; 2 Shandong Institute of Metrology, Jinan 250014, China; 3 School of Chemical Engineering and Materials, Changzhou Institute of Technology, 666 Liaohe Road (S), Changzhou 213022, China; 4 State Key Laboratory of Materials-Oriented Chemical Engineering, College of Chemical Engineering, Nanjing Tech University, 30 Puzhu Road (S), Nanjing 211816, China

**Keywords:** trimetallic FeCoNi-MOF, peroxidase-like activity, nanozyme-like colorimetric sensor, thiophenol, environmental water samples

## Abstract

In recent years, nanozymes have attracted particular interest and attention as catalysts because of their high catalytic efficiency and stability compared with natural enzymes, whereas how to use simple methods to further improve the catalytic activity of nanozymes is still challenging. In this work, we report a trimetallic metal–organic framework (MOF) based on Fe, Co and Ni, which was prepared by replacing partial original Fe nodes of the Fe-MOF with Co and Ni nodes. The obtained FeCoNi-MOF shows both oxidase-like activity and peroxidase-like activity. FeCoNi-MOF can not only oxidize the chromogenic substrate 3,3,5,5-tetramethylbenzidine (TMB) to its blue oxidation product oxTMB directly, but also catalyze the activation of H_2_O_2_ to oxidize the TMB. Compared with corresponding monometallic/bimetallic MOFs, the FeCoNi-MOF with equimolar metals hereby prepared exhibited higher peroxidase-like activity, faster colorimetric reaction speed (1.26–2.57 folds), shorter reaction time (20 min) and stronger affinity with TMB (2.50–5.89 folds) and H_2_O_2_ (1.73–3.94 folds), owing to the splendid synergistic electron transfer effect between Fe, Co and Ni. Considering its outstanding advantages, a promising FeCoNi-MOF-based sensing platform has been designated for the colorimetric detection of the biomarker H_2_O_2_ and environmental pollutant TP, and lower limits of detection (LODs) (1.75 μM for H_2_O_2_ and 0.045 μM for TP) and wider linear ranges (6–800 μM for H_2_O_2_ and 0.5–80 μM for TP) were obtained. In addition, the newly constructed colorimetric platform for TP has been applied successfully for the determination of TP in real water samples with average recoveries ranging from 94.6% to 112.1%. Finally, the colorimetric sensing platform based on FeCoNi-MOF is converted to a cost-effective paper strip sensor, which renders the detection of TP more rapid and convenient.

## 1. Introduction

Thiophenol (TP) is an essential industrial raw material which is widely used as an intermediate or auxiliary in the fields of medicine, pesticides, polymer materials and organic synthesis [1,2,3]. However, massive amounts of TP discharged from industrial waste water and waste gas cause serious pollution to environmental water bodies and soil and pose a great threat to ecological security. If humans are exposed to TP, serious damage will be caused to their immune system, nervous system and respiratory system. Even death can be caused [2,4]. Therefore, an accurate, sensitive and convenient method to detect TP is urgently needed.

A variety of analytical methods have been developed so far to detect H_2_O_2_ and TP, such as high-performance liquid chromatography (HPLC) [5], gas chromatography (GC) [6], gas chromatography–mass spectrometry (GC-MS) [7], nonlinear optical spectroscopy [8], electrochemical sensors [9], fluorescent sensors based on nanomaterials [10,11,12] or synthetic probes [13,14,15] and so on. In Zribi’s work, sensors based on silver nanoplates (Ag NPTs), colloidal solutions coupled with cyclic voltammetry (CV) and linear sweep voltammetry (LSV) analyses were applied for the detection of H_2_O_2_, and a low limit of detection (LOD) of 16 µM and a wide linear range of 0–1000 µM were obtained. And in Wu’s work, a nanofibrous membrane-based fluorescent and colorimetric sensor was synthesized for the convenient and portable detection of thiophenols relying on the nucleophilic substitution reaction of chlorinated dipyrrole fluoride (BODIPY). However, these methods have their shortcomings. For example, although chromatography is sensitive, it requires bulky equipment and complicated and time-consuming detection processes, which can hardly meet the needs of field detection [16]. Some detection methods based on fluorescence are relatively convenient, but they are generally limited by the complex synthesis process of the fluorescent probe [4]. Analysis based on colorimetric sensors can overcome the shortcomings of the above detection methods and achieve qualitative detection of TP by only simple visual observation, which satisfyingly meets the need for field detection. However, there are very few studies concentrating on the colorimetric detection of TP, and most of the colorimetric detection methods involve a complex synthesis process of colorimetric probes, which makes the detection lack practicality. Colorimetric sensors based on nanozymes with peroxidase-like activity are expected to emerge as a new colorimetric method for TP detection due to their simple detection principles, high sensitivity, rapidity and convenience [17]. The major detection principle of nanozyme colorimetric sensors is that the nanozymes with peroxidase-like activity oxidize the chromogenic substrate 3,3,5,5-tetramethylbenzidine (TMB) in the presence of H_2_O_2_ into its oxidation state oxTMB, which is blue, and the target will fade or deepen the color of the detection system through reduction, adsorption or other ways, so as to realize the detection of the target.

Nanozymes are a group of artificially synthesized nanomaterials with biological enzyme activity such as peroxidase-like activity, oxidase-like activity, catalase-like activity and so on. Compared with natural enzymes, nanozymes possess remarkable advantages including high catalytic activity, simple preparation process, low cost, stability and wide pH and temperature application range [18,19]. Some nanomaterials such as platinum nanoparticles [20], Co_3_O_4_ [21], carbon dots [22] and metal–organic frameworks (MOFs) [23,24] show peroxidase-like activity, among which MOFs have received extensive attention due to their simple preparation method, versatility and adjustability [7,25,26,27,28]. Some MOF-based nanozymes have been applied to the construction of colorimetric sensors to detect glutathione, cysteine and some other biological molecules [29,30,31], but there is hardly any research reporting the detection of the environmental pollutant TP so far. As a result, it is of great significance to construct colorimetric sensors with nanozymes to achieve the quick, sensitive and convenient detection of TP.

In addition, how to further improve the catalytic activity of MOF-based nanozymes has also become the focus of research in recent years. For MOF-based nanozymes, their catalytic activity is closely related to their metal centers. So, the introduction of multifunctional metal centers or functional groups has been involved as an important method to improve their activity [32]. In previous reports, the original metal nodes of the MOF are partially replaced by other metal nodes to form bimetallic MOFs with greatly enhanced catalytic properties, such as reported Co/Fe MOF, Co/Mn MOF and so on [33,34,35,36,37]. However, few reports doped two elements in the initial MOF to form trimetallic MOF nanozymes to construct colorimetric sensors. We hope to improve the catalytic activity of MOF-based nanozymes significantly by doping two elements in the original MOF to design a trimetallic MOF. Lu et al. proved the splendid synergistic electron transfer effect between different metals using a maximized-entropy approach. Their work also showed that polymetallic MOFs have higher catalytic activity than single-metal MOFs containing equimolar metals [38]. Therefore, based on the similar peroxidase activity, extranuclear electron arrangement and other physicochemical properties of Fe, Co and Ni [33,34,37,39], Fe/Co/Ni-based trimetallic MOFs are synthesized by replacing the original Fe nodes of the Fe-MOF partially with Co and Ni nodes (when Fe, Co and Ni coordinate with ligands, in order to make the coordination environment and probability roughly the same, Fe^3+^ used in the synthesis of MIL-53 (Fe) was replaced with Fe^2+^).

In this work, FeCoNi-MOF with both oxidase-like and peroxidase-like activity was synthesized. And corresponding monometallic Fe, Co and Ni MOFs as well as bimetallic FeCo, FeNi and CoNi MOFs were prepared for comparison and all monometallic/bimetallic MOFs contain equimolar metals with FeCoNi-MOF. The crystal structure, morphology and element composition of seven MOFs were characterized. Additionally, their peroxidase-like activities were determined and compared. The steady-state kinetics with H_2_O_2_ and TMB as the substrates and catalytic mechanisms of the activation of H_2_O_2_ on the nanozyme-like material FeCoNi-MOF were systematically investigated. Furthermore, based on the peroxidase-like activity of FeCoNi-MOF, a colorimetric sensor was further constructed and applied to the detection of TP in real water samples.

## 2. Results and Discussion

### 2.1. Characterization of Seven MOFs

**Figure 1:**
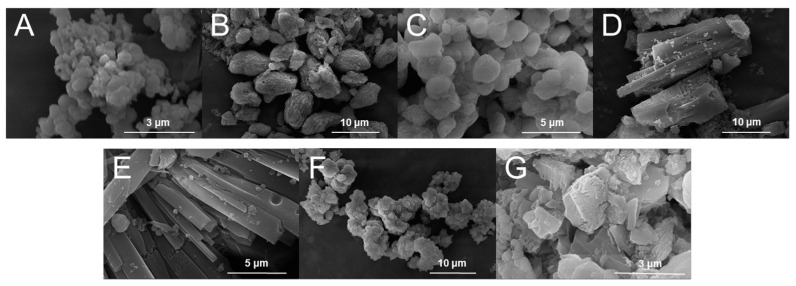
FESEM images of (**A**) Fe-MOF, (**B**) Co-MOF, (**C**) Ni-MOF, (**D**) FeCo-MOF, (**E**) FeNi-MOF, (**F**) CoNi-MOF, (**G**) FeCoNi-MOF materials.

The synthesized MOF materials are shown in Figure S1. The SEM images of seven MOFs (Fe-MOF, Co-MOF, Ni-MOF, FeCo-MOF, FeNi-MOF, CoNi-MOF and FeCoNi-MOF) are shown in Figure 1. The MOFs with different metal elements exhibit irregular prismatic, spherical or blocky morphologies. Trimetallic FeCoNi-MOF looks like irregular blocky particles with layered surface structure. The formation of the layered structure on the surface is conducive to improving the catalytic activity of FeCoNi-MOF owing to the large reaction contact area and high proportion of exposure of active sites. As shown in the SEM images of seven MOFs, not all of these seven MOFs are nanomaterials. In order to describe them more accurately and rigorously, they are referred to as nanozyme-like materials collectively in the following text.

The field-emission scanning electron microscopy (FESEM) and elemental mapping images (Figures S2–S8) of seven MOFs confirmed that all of the constituent elements (C, O and metal elements) are evenly distributed throughout the whole material. And the composition of the elements in MOFs was analyzed by SEM-EDS mapping, as shown in Figure S9. The metal element ratio in bimetallic and trimetallic MOFs is close to 1:1 and 1:1:1.

**Figure 2:**
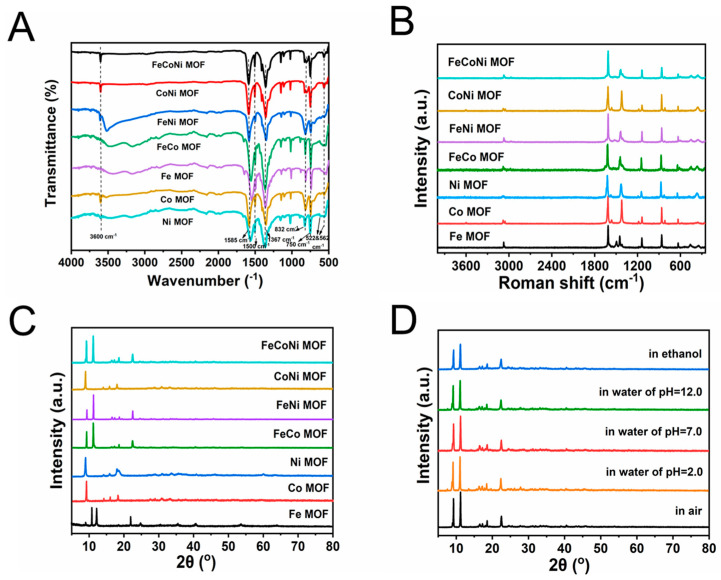
(**A**) FT-IR spectra, (**B**) Raman spectra and (**C**) XRD patterns of seven MOFs. (**D**) XRD patterns of FeCoNi-MOF after immersion under different conditions.

The crystal structure of seven MOFs was further confirmed using X-ray diffraction, FT-IR spectra and Raman spectroscopy. As revealed in Figure 2A,B, the absorption peak of the M(μ2–OH)M (M stands for metals) group at 832 cm^−1^ and Raman bands at 1417 cm^−1^ and 1610 cm^−1^ can be clearly observed, which indicates the successful synthesis of seven MOFs and the consistency of the structures of seven MOFs. Additionally, strong XRD peaks also show the successful synthesis of seven MOFs with distinct crystal structure (Figure 2C). Co-MOF and Ni-MOF were synthesized by Co^2+^ or Ni^2+^ coordinating with BDC ligands, and Co-BDC was isostructural to the previously reported Ni-BDC (No. 985792, space group of C2/m, Cambridge Crystallographic Data Center) [40]. Their XRD patterns are consistent with those reported in previous literature [41], and there are two characteristic XRD peaks at 9.5° and 18°, ascribed to the crystal facets of (200) and (400), which illustrate the successful synthesis of Co-MOF and Ni-MOF. Note that in order to make the same coordination environment and probability as Co^2+^ and Ni^2+^ during the synthesis process of MOFs, Fe^2+^ was used for synthesizing Fe-MOF instead of Fe^3+^. Thus, synthesized Fe-MOF is a mixed state with both MIL-53(Fe) (XRD peaks at 12° and 22.5°) and Fe^2+^-BDC structures (XRD peaks at 9.27°, 11.1° and 25.32°), which is not exactly the same as MIL-53(Fe) [42,43]. Additionally, XRD patterns of bimetallic/trimetallic MOFs are a combination of the XRD patterns of monometallic MOFs.

The stability of MOFs in aqueous solutions with different pH values and in organic solvents plays a crucial role in the construction of nanozyme colorimetric sensors. Hence, the chemical stability of the seven MOFs was tested, while FeCoNi-MOF was selected as the representative material and the results are presented in Figure 2D. No obvious changes can be observed in the XRD pattern of FeCoNi-MOF after it was immersed in aqueous solution with pH 2.0, 7.0 and 12.0 as well as methanol for 24 h. Similar results were obtained for the other six MOFs. As a result, the seven materials were found to be stable in organic solvents and aqueous solution at pH values of 2.0, 7.0 and 12.0, clearly certifying that they are stable enough to construct the sensor.

The N_2_ adsorption–desorption isotherm is shown in Figure S10, and it is used to determine the surface areas of the seven MOFs. The Fe MOF has the highest BET surface area of 114.9 m^2^ g^−1^, and the specific surface area order of other six MOFs is FeNi-MOF (98.76 m^2^ g^−1^) > FeCoNi-MOF (70.03 m^2^ g^−1^) > Ni-MOF (60.72 m^2^ g^−1^) > CoNi-MOF (58.44 m^2^ g^−1^) > Co-MOF (47.94 m^2^ g^−1^) > FeCo-MOF (35.27 m^2^ g^−1^). There is no significant relationship between the specific surface area of these seven materials and their element variety as well as catalytic activity. 

**Figure 3:**
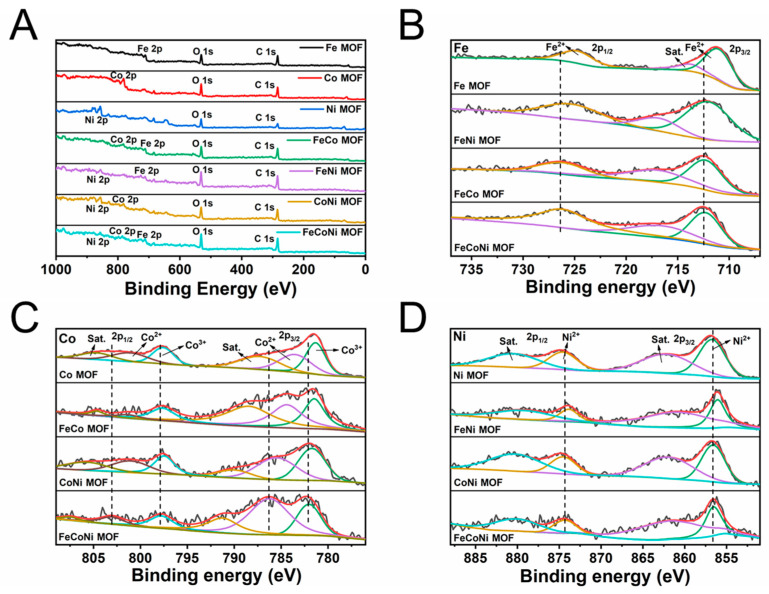
XPS images of the seven MOFs: (**A**) the survey spectra of seven MOFs; the high-resolution XPS spectrums of (**B**) Fe 2p, (**C**) Co 2p, and (**D**) Ni 2p.

Furthermore, XPS was used to analyze the valence of the elements in seven MOFs and investigate the origin of the synergistic effect in the polymetallic MOF. The full survey spectra of seven MOFs are shown in Figure 3A. The high-resolution spectra of Fe, Co and Ni are shown in Figure 3B–D, which were deconvoluted to verify the oxidation states. In the high-resolution (HR) XPS spectra of Fe 2p (Figure 3B) of four MOFs containing Fe, two obvious binding energy peaks at around 711.5 eV and 725.5 eV are ascribed to Fe 2p_3/2_ and Fe 2p_1/2_, respectively, demonstrating that only the 2+ oxidation state of iron exists in the four MOFs [44]. The HR-XPS spectra of Co 2p of four MOFs containing Co (Figure 3C) exhibit binding energy peaks at around 781.4 eV and 783.7 eV for Co 2p_3/2_ and 797.5 eV and 801.0 eV for Co 2p_1/2_, respectively, supporting the coexistence of 2+ and 3+ oxidation states of cobalt in four MOFs [45]. The coexistence of 2+ and 3+ states of cobalt can facilitate the electron transfer between the FeCoNi-MOF and the TMB, which is believed to be the reason that the FeCoNi-MOF has oxidase activity [46]. As for Ni 2p, the binding energy peaks at 856.8 eV and 874.6 eV displayed in Figure 3D correspond with Ni 2p_3/2_ and Ni 2p_1/2_, respectively, indicating that in four MOFs containing Ni, only the 2+ oxidation state of Ni exists [47]. Interestingly, the characteristic peaks of Fe 2p, Co 2p and Ni 2p shift to higher binding energies for FeCoNi-MOF, which may originate from the change in the coordination environment of the metal centers caused by the introduction of Co and Ni in the Fe-MOF [38]. It also shows that FeCoNi-MOF has elevated oxidation states and thus higher oxidative potentials to catalyze the activation of H_2_O_2_ [38].

### 2.2. The Peroxidase-like Activity of Seven MOFs

The peroxidase-like activity of seven MOFs is further evaluated (Table S1). An obvious absorbance change at 652 nm and the appearance of dark blue can be observed only in the presence of TMB, H_2_O_2_ and one of seven MOFs, demonstrating the intrinsic peroxidase-like activity of seven MOFs.

The system containing only FeCoNi-MOF and TMB also had an appearance of very slight blue, indicating that the seven MOFs have oxidase activity and can oxidize TMB directly. The mechanism showing peroxidase-like activity of seven MOFs (taking CoNi-MOF and FeCoNi-MOF as examples) is displayed in Scheme S1. The oxidase-like activity of the seven MOFs is basically similar and the peroxidase-like activity of the seven MOFs differs with the constituent elements (Table S1). Trimetallic FeCoNi-MOF has the strongest peroxidase activity. In addition, the peroxidase activity of bimetallic MOF is significantly higher than that of monometallic MOF with the activity order of the seven materials being FeCoNi-MOF > FeCo-MOF > FeNi-MOF > CoNi-MOF > Fe-MOF > Co-MOF > Ni-MOF.

Therefore, FeCoNi-MOF is chosen to construct the nanozyme-like sensor in order to achieve high sensitivity. Because all MOFs contain equimolar metals, the synergy between two metals and/or three metals plays an important role in improving peroxidase activity.

### 2.3. Optimization of Nanozyme-like Colorimetric Sensor We Constructed

In order to make the sensor possess high detection sensitivity, some key parameters were optimized. The optimization details are presented in the Supporting Information and the results are shown in Figure S11.

As a result, the conditions of sensor construction in the following experiments are summarized as follows: 25 μg mL^−1^ and 500 μM as the concentrations of the suspension of FeCoNi-MOF and TMB in the system, respectively, 4.0 as the system pH, 20 min as the reaction time and 40 °C as the reaction temperature. The optimization details are displayed in the Supporting Information.

### 2.4. The Sensitivity of H_2_O_2_ Detection

The concentration of H_2_O_2_ in the system was also optimized. The LOD of H_2_O_2_ is calculated to be 1.75 μM (3SB/k) and the linear range is between 6 μM and 800 μM with the regression equation of y = 0.001x + 0.666 and R^2^ is 0.996 (where y and x represent absorbance at 652 nm and the concentration of H_2_O_2_ in the system, respectively). Moreover, to obtain a wide detection range and accurate detection results, the absorbance of the system at 652 nm is expected to be around 1.0. Therefore, the concentration of H_2_O_2_ is set to 400 μΜ in the following experiments. The specific discussion is shown in the Supporting Information (Figures S12 and S13, Table S2).

### 2.5. Investigation of the Catalytic Mechanism of H_2_O_2_ Activation on FeCoNi-MOF and Identification of the Species of Free Radicals

**Figure 4:**
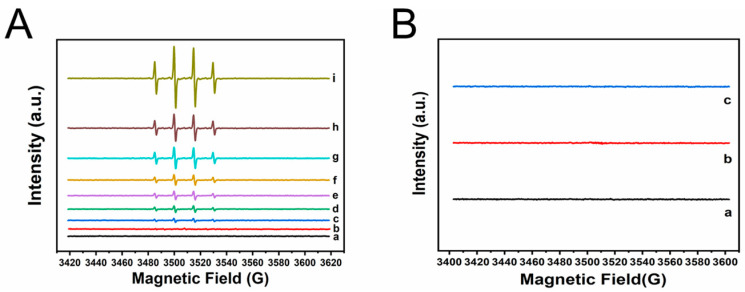
The test of the radical species. (**A**) EPR spectra of the activation of H_2_O_2_ under different conditions with DMPO as the spin-trapping agent: (a) DMPO only; (b) DMPO+H_2_O_2_; (c) DMPO+H_2_O_2_+Ni-MOF; (d) DMPO+H_2_O_2_+Co-MOF; (e) DMPO+H_2_O_2_+Fe-MOF; (f) DMPO+H_2_O_2_+CoNi-MOF; (g) DMPO+H_2_O_2_+FeNi-MOF; (h) DMPO+H_2_O_2_+FeCo-MOF; (i) DMPO+H_2_O_2_+FeCoNi-MOF; (**B**) EPR spectra of activation of H_2_O_2_ under different conditions with TEMP as the spin-trapping agent: (a) TEMP only; (b) TEMP+H_2_O_2_; (c) TEMP+H_2_O_2_+FeCoNi-MOF.

To investigate the catalytic mechanism of H_2_O_2_ activation on FeCoNi-MOF, EPR experiments were conducted to verify species of free radicals (Figure 4A,B). With DMPO as the trapping agent, the coexistence of MOFs and H_2_O_2_ results in characteristic peaks of DMPO^•^–OH (with the hyperfine coupling constants of a_N_ = 15.01 G and a_β-H_ = 14.70 G) [48,49]. The results demonstrate the formation of ·OH in the reaction systems clearly. Furthermore, the intensity of the EPR signals of different materials is compared. The intensity of the EPR signal of FeCoNi-MOF is the strongest, while the intensity of the EPR signals of bimetallic FeCo, FeNi and CoNi-MOF is stronger than that of monometallic Fe, Co and Ni MOFs, which is consistent with the order of their activity. These results indicate that the synergy between two metals and even three metals plays an important role in the process of activating H_2_O_2_ [38]. Notably, with TEMP as the radical trapping agent, no distinct EPR signals are observed (see Figure 4B), reflecting that superoxide radicals (·O_2_^−^) are not involved in the reaction system.

### 2.6. The Steady-State Kinetic Analysis with FeCoNi-MOF for the Activation of H_2_O_2_

**Figure 5:**
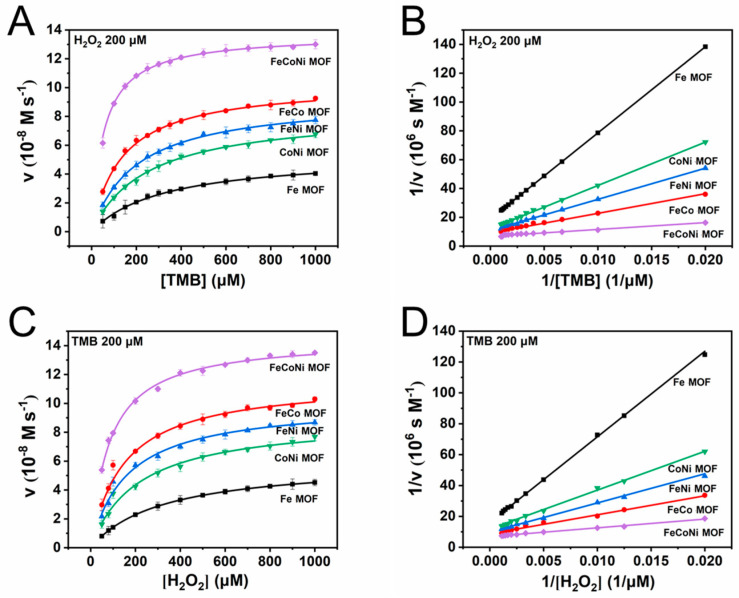
Steady-state kinetic assay of five representative MOFs as the catalyst with H_2_O_2_ and TMB as the substrate: (**A**) Michaelis–Menten curves of five MOFs with the concentration of H_2_O_2_ fixed at 200 μM; (**B**) double reciprocal plots of five MOFs with the concentration of H_2_O_2_ fixed at 200 μΜ; (**C**) Michaelis–Menten curves of five MOFs with the concentration of TMB fixed at 200 μM; (**D**) double reciprocal plots of five MOFs with the concentration of TMB fixed at 200 μΜ.

The Michaelis–Menten equation is often used to describe the steady-state kinetics of HRP or some nanozymes [18,50,51]. Hence, we hypothesize that the processes of TMB oxidation via H_2_O_2_ activation on seven MOFs also obey the principle. To verify the hypothesis, steady-state kinetic measurements of TMB oxidation were carried out at pH 4.0 with representative Fe-MOF, FeCo-MOF, FeNi-MOF, CoNi-MOF and FeCoNi-MOF as the nanozyme-like materials, and the results are shown in Figure 5A–D. As is shown in Figure 5A, the concentration of H_2_O_2_ was fixed at 200 μΜ, and with the initial concentration of TMB increasing from 50 μΜ to 1000 μΜ, the initial rate of the catalytic reaction increases gradually. The experimental data are fitted with the Michaelis–Menten equation, and the results show that the catalytic process can be well described by the Michaelis–Menten model. In order to obtain the corresponding V_max_ and K_m_ values with different materials to the catalyst, the Michaelis–Menten curves are converted to double reciprocal curves (Figure 5B), and the obtained K_m_ values, V_max_, double reciprocal equations and R^2^ are listed in Table 1. When the FeCoNi-MOF is used as the catalyst, the smallest K_m_ value (0.054 mM) and the largest V_max_ (13.68 × 10^−8^ M s^−1^) are obtained, and the performances of the three bimetallic MOFs are better than that of monometallic Fe-MOF. In parallel, when the concentration of TMB was fixed at 200 μΜ, the experimental data can also be well fitted with the Michaelis–Menten equation (Figure 5C), and the K_m_ values, V_max_, double reciprocal equations and R^2^ obtained from the double reciprocal curves (Figure 5D) are shown in Table 2. Similarly, the obtained K_m_ value is the smallest (0.082 mM) and the V_max_ is the largest (14.43 × 10^−8^ M s^−1^) with FeCoNi-MOF as the catalyst. Compared with Fe-MOF, bimetallic FeCo-MOF, FeNi-MOF and CoNi-MOF all show better performance. These results indicate that the trimetallic FeCoNi-MOF displays a 2.50–5.89 times stronger affinity for TMB as well as a 1.73–3.94 times stronger affinity for H_2_O_2_ than the other four MOFs. Additionally, compared with three bimetallic and one monometallic MOF, FeCoNi-MOF has 1.33–2.57 times faster reaction kinetics with TMB as the varying substrate and 1.26–2.41 times faster reaction kinetics with H_2_O_2_ as the varying substrate. This observation further verifies that the synergistic effect between Fe, Co and Ni can significantly enhance the catalytic activity of the nanozyme-like materials and promote the activation of H_2_O_2_.

In order to explore the reaction mechanism of FeCoNi-MOF activating H_2_O_2_ to oxidize TMB, the concentration of the fixed substrates was changed to obtain different Michaelis–Menten kinetic curves. With initial H_2_O_2_ concentrations fixed at 50, 100, 200 and 500 μΜ, the average values of K_m_ and V_max_ are 0.061 mM and 13.32 × 10^−8^ M s^−1^, respectively (Figures S14 and S15, Table 3). In parallel, with initial TMB concentrations fixed at 50, 100, 200 and 500 μΜ, the average values of K_m_ and V_max_ obtained are 0.074 mM and 14.30 × 10^−8^ M s^−1^, respectively (Figures S16 and S17, Table 3). In comparison with K_m_ and V_max_ values of HRP and nanozymes from references (see Tables S3 and S4), smaller K_m_ values (1.35–174.19 folds) and much larger V_max_ (~215.36 folds) are obtained for FeCoNi-MOF prepared in this study, demonstrating the stronger affinity of FeCoNi-MOF for TMB and H_2_O_2_, faster reaction kinetics and, hence, shorter reaction time (20 min) of TMB oxidation on FeCoNi-MOF than that on HRP and other nanozymes. These results clearly verify that FeCoNi-MOF is promising for constructing colorimetric sensors to detect TP in environmental water samples.

The ping-pong mechanism is a reaction mechanism generally followed by natural enzymes and nanozymes, which means that substrates and products are alternately combined with or released from enzymes. In order to determine whether the catalytic reaction process of FeCoNi-MOF with H_2_O_2_ and TMB as substrates conforms to this mechanism, the double reciprocal plots obtained with different concentrations of fixed TMB or H_2_O_2_ are compared in Figure S18. The lines are parallel, indicating that the catalytic process based on FeCoNi-MOF also follows the ping-pong mechanism [18,52]. These results imply that initially, H_2_O_2_ is activated on FeCoNi-MOF to generate active radicals, which subsequentially react with TMB to produce blue oxTMB.

### 2.7. The Sensitivity and Possible Mechanism of TP Detection

**Figure 6:**
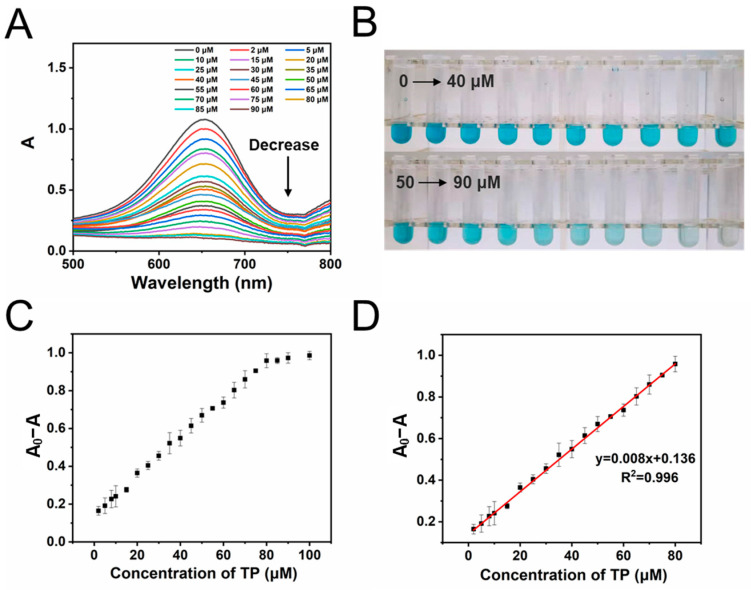
(**A**) The UV absorption spectra of different systems with different concentrations of TP added (0–90 μM); (**B**) corresponding optical photograph of different reaction systems with different concentrations of TP (0–90 μM); (**C**) scatter plot (absorbance at 652 nm) for TP detection using a nanozyme-like sensor with FeCoNi-MOF as the catalyst in the range of 0–100 μΜ; (**D**) linear calibration plot (absorbance at 652 nm) for TP detection using a nanozyme-like sensor with FeCoNi-MOF as the catalyst in the range of 0.5–80 μM.

Given the highly effective activation of H_2_O_2_ on FeCoNi-MOF, the colorimetric sensor was further constructed to detect TP with concentrations ranging from 0 μM to 100 μM (Figure 6). Increasing the TP concentration from 0 μM to 100 μM leads to gradual fading of the color of the reaction system, and the absorbance at 652 nm decreases (see Figure 6A,B). When the concentration of TP is below 80 μM, there is a good linear relationship with an R^2^ of 0.998 between the change in the absorbance at 652 nm and the concentration of TP, which is shown as follows (Figure 6C,D),

(A_0_ − A_x_) = 0.010C_x_ + 0.140

where A_0_ is the initial absorbance from oxTMB without TP, and A_x_ is the absorbance in the presence of TP with a concentration of C_x_.

The linear range of this sensor for the detection of TP is between 0.5 μM and 80 μM (see Figure 6D) with the LOD and LOQ calculated to be 0.045 μM (3S_B_/k) and 0.150 μM (10S_B_/k), respectively. Compared with other methods reported in studies [53,54,55,56,57,58,59,60,61], the newly constructed colorimetric sensor shows a much lower LOD and wider linear range, demonstrating that the sensor with excellent detection ability and high sensitivity is ready for broad application (Table S5). We also tested the cyclic stability of the FeCoNi MOF, and the results show that it has good reusability over a six-cycle run (Figure S19). To avoid potential absorbance artifacts arising from the concentrated, colored MOFs, before each absorbance test, the materials were separated from the system with the filter membrane. (Reaction conditions: 2 mg mL^−1^ FeCoNi MOF, 500 μM TMB, 500 μM H_2_O_2_, pH 4.0, 20 min, under 40 °C in 5 mL reaction system.) In addition, the sensor has good repeatability (relative standard deviations (RSDs) (n = 3) 3.39%) and reproducibility (RSD of interday (n = 3) 5.26%, intraday (n = 3) 6.09%) (Table S6).

For the detection of TP, the characteristic functional sulfhydryl group (-SH) on TP plays an important role. The principle of detecting TP was explored by adjusting the order of adding the suspension of FeCoNi-MOF, TMB, H_2_O_2_ and TP. In system 1, TP was added to the system first, and TMB, H_2_O_2_ and the suspension of FeCoNi-MOF were then added in sequence. The color of the system faded after incubation at 40 °C for 20 min. This indicates that TP may react with the active free radicals produced by H_2_O_2_ activation on FeCoNi-MOF and consume some free radicals, so that a small amount of TMB is oxidized by free radicals, making the color of the system lighter. In system 2, TMB, H_2_O_2_ and the suspension of FeCoNi-MOF were added in sequence to the system and the system was incubated at 40 °C for 20 min. TP was added finally, and the color of the system faded similarly. This indicates that after TMB completely converts to blue oxTMB, TP will reduce oxTMB back to colorless TMB. Finally, the absorbance at 652 nm of system 1 and system 2 is the same (about 0.4) (Figure S20). According to the fading phenomenon, the principle of the sensor to detect TP might be described as follows. Firstly, TP can reduce blue oxTMB to colorless TMB by hydrogen donation of the -SH. Secondly, TP competes with TMB for ·OH, and it will react with ·OH before TMB due to its high activity, which leads to the low yield of the blue oxTMB. And these two ways coexist in the actual experiments. The consumption of free radicals by TP can also be proven by EPR experiments. As shown in Figure S21, when TP or TMB is added to the reaction system, the EPR signal becomes much weaker, attributed to the consumption of free radicals by TP or TMB, illustrating that the competitive effect of TP and TMB for the free radicals is an important mechanism for detecting TP. The principles we speculated are basically the same as the principles of detecting other sulfhydryl compounds by nanozyme sensors, and the changes in the form of TMB in the whole detection process are discussed in the Discussion Section of the Supporting Information (Figure S22) [16,62,63].

Additionally, the detection of TP was not interfered with by common inorganic ions (Fe^3+^, NH_4_^+^, Ni^2+^, Co^2+^, HCO_3_^−^, PO_4_^3−^, Ac^−^, Br^−^) and molecular organic pollutants (BPA, PFOA, PFOS, 2,4-D, P, 4-OP, 4-NP, 2,4-CP and SD) (Figures S23 and S24), and more details about the selectivity of the nanozyme-like sensor are discussed in the Supporting Information.

### 2.8. Colorimetric Detection of TP in Real Water Samples

The newly built nanozyme-like colorimetric sensor with FeCoNi-MOF as the catalyst was applied for the detection and analysis of TP in real water samples including tap water, Tai Lake water, Xuanwu Lake water and Jiuxiang River water. The physical and chemical water quality indicators of the distilled water and four real water samples including pH, total dissolved solids (TDS), salinity (PSU) and total organic carbon (TOC) are listed in Table S7. The typical UV spectrum curves of four water samples are displayed in Figure S25A–D, and the analytical results of four real water samples are given in Table 4. No TP was found in four water samples. In addition, comparisons between the added TP and the detected TP as well as the spiked recoveries are used to evaluate the reliability and sensitivity of the sensor. With the spiked concentrations of 2, 5, 10 and 20 μM, the average spiked recoveries of the four samples are 94.6–112.1%, with the RSD ranging from 0.11% to 7.32%, demonstrating that PSU, TOC and TDS at high concentrations in natural water have little effect on the detection of TP. As a result, this novel nanozyme-like colorimetric sensor based on FeCoNi-MOF is adaptable for the rapid analysis of TP in real water samples.

### 2.9. The Feasibility of Paper Strip Sensors

**Figure 7:**
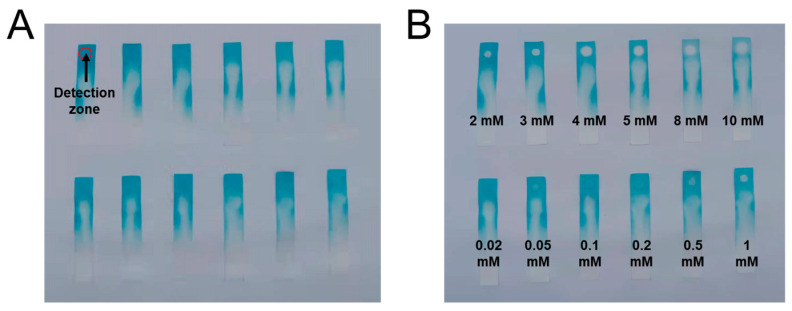
Activity of paper strip sensor using different concentrations of TP (2 µL, 0.02–10.00 mM): (**A**) optical photographs of standard paper strip sensors after soaked in the standard reaction system for 30 min and dried at room temperature for 10 min; (**B**) optical photographs of paper strip sensors with a drop of GSH solution (0.02–10 mM, 2 μL) added on them.

In order to make TP detection more convenient, a paper strip sensor was attempted, and the results are shown in Figure 7 and Figure S26. Briefly, 2 μL of TP solution (10 μM) with different concentrations (0.02–10 mM) was dropped on the detection zone of the paper strip sensor, and the blue color of the detection zone quickly turned colorless (see Figure 7A,B). Furthermore, increasing the TP concentration from 0.05 mM to 10 mM led to more remarkable fading. Three repeated paper strip tests were performed. The color of the paper strip sensor was evaluated by grayscale quantization values, and then analyzed by the software ImageJ_v1.8.0 (Figure S27, take one of the paper strip sensors as the example). The grayscale quantization values of three paper strip sensors are summarized in Table S8. When the concentration of thiophenol decreases below 0.2 mM, the grayscale quantization values appear to have irregular changes. The possible interferences of other representative pollutants with a concentration of 0.5 mM on the paper strip sensor were also investigated, and the results are shown in Figure S26B. The results obtained are similar to those obtained only using TP solution, suggesting that the paper strip sensor is not interfered with by other pollutants and is suitable for detecting TP. Additionally, the duration that the paper strip sensor remains active is of vital importance for the application of the paper sensor. Figure S26A presents the duration of the paper sensor for TP detection. At a TP concentration of 0.5 mM, the paper strip sensor remains active within 60 min, highlighting that the FeCoNi-MOF-based paper strip sensor can be used as a rapid, convenient and reliable method for TP detection.

## 3. Experimental Procedures

### 3.1. Preparation of Seven MOFs

FeCoNi-MOF and other monometallic and bimetallic materials (Fe-MOF, Co-MOF, Ni-MOF, FeCo-MOF, FeNi-MOF and CoNi-MOF) used for comparison were synthesized according to references with some modifications [38,64]. The specific synthesis details are described in the Supporting Information. And the detailed information of chemicals and equipments are all in the Supporting Information. 

### 3.2. Peroxidase-like and Oxidase-like Activity Measurement

The catalytic oxidation of colorless TMB to blue oxTMB in the presence or absence of H_2_O_2_ was used to evaluate the peroxidase-like or oxidase-like activities of the FeCoNi-MOF and the other six materials for comparison. Acetate buffer (0.1M) with pH 4.0 was selected as the matrix in the evaluation process [51]. Each of the seven materials was added to the acetate buffer (pH 4.0) to form seven suspensions of 500 μg mL^−1^. Then, 50 μL of the suspension was dispersed into 850 μL acetate buffer (0.1 M, pH 4.0), to which 50 μL of H_2_O_2_ (10 mM) and 50 μL TMB (10 mM in DMF) were added. The absorbance of the mixture was recorded at 652 nm after it was incubated at 40 °C for 30 min to complete a thorough conversion of TMB to oxTMB. The material which had the highest peroxidase activity was chosen to construct the colorimetric sensor.

The effect of some factors on the detection sensitivity of the nanozyme-like sensor was studied and the parameters were optimized.

### 3.3. Steady-State Kinetic Analysis of H_2_O_2_ Activation on Co(BDC)TED_0.5_

For natural horseradish peroxidase (HRP), the enzymatic reaction kinetics with TMB and H_2_O_2_ as the substrates can be well described by the Michaelis–Menten equation (see Equation (1)) [18]:

V = V_max_ [S]/(K_m_ + [S]) (1)

1/V = K_m_/V_max_·1/[S] + 1/V_max_ (2)

where K_m_ is the Michaelis–Menten constant, V_max_ is the reaction rate when the enzyme is saturated with the substrate, [S] is the concentration of the substrate, and V is the initial rate of the reaction when the substrate concentration is [S].

Hence, we speculated that the steady-state kinetic processes of the catalytic reaction with seven MOFs can be fitted with the Michaelis–Menten equation. The steady-state kinetics measurements with H_2_O_2_ and TMB as the substrates were used to verify our speculation.

### 3.4. The Identification of the Radical Species and the Determination of the Contribution Ratio

The EPR measurements with DMPO and TEMP as the spin-trapping agents were carried out to verify the radical species produced in the catalytic activation of H_2_O_2_ on FeCoNi-MOF (FeCoNi-MOF: 25 μg mL^−1^; DMPO/TEMP: 200 μM; H_2_O_2_: 400 μM; system pH: 4.0; spin-trapping time: 10 min; spin-trapping temperature: 40 °C; system volume: 1 mL). If multiple radical species are involved in the reaction system, tests using radical scavengers were further conducted to determine the ratio of different radicals [65].

### 3.5. Detection of TP with FeCoNi-MOF as Peroxidase and the Selectivity of This Colorimetric Sensor

TP detection was carried out by adding 50 μL TP solution with different concentrations (0–2 mM) into the reaction system (FeCoNi-MOF 25 μg mL^−1^, TMB 0.5 mM and H_2_O_2_ 0.5 mM) under optimal conditions. Moreover, the selectivity of this colorimetric sensor was also evaluated.

### 3.6. Calculation of the Limit of Detection (LOD) and Limit of Quantification (LOQ)

The limit of detection (LOD) and limit of quantification (LOQ) were calculated according to Equations (3) and (4) [65]:

LOD = 3S_B_/k (3)

LOQ = 10S_B_/k (4)

where S_B_ is the standard deviation of three repeated measurements of the blank samples, and k is the slope of the regression equation when the target analyte is in the linear concentration range.

### 3.7. Practical Application of This Newly Built Sensor in Real Environmental Samples

Four real environmental water samples (tap water, Tai Lake water, Xuanwu Lake water and Jiuxiang River water) and the samples spiked with four standard TP solutions (2 μM, 5 μM, 10 μM and 20 μM) were tested separately using the sensor (FeCoNi-MOF: 25 μg mL^−1^; TMB: 500 μM; H_2_O_2_: 400 μM; system pH: 4.0; reaction time: 20 min; reaction temperature 40 °C; system volume: 1 mL). For the spiked samples, comparisons between the added amount and the average detected amount as well as the average recoveries were used to evaluate the reliability of the colorimetric sensor.

### 3.8. Fabrication of Paper Strip Sensor

To make the colorimetric sensor more convenient and suitable for the rapid detection of TP in environmental water samples, we prepared paper strip sensors. Briefly, Axiva ashless filter paper (no. 420 R, pore size of 5 μm) was used to prepare paper strip sensors. The filter paper strips were soaked in the standard reaction system (system volume 2 mL, TMB 500 μM, H_2_O_2_ 500 μM and FeCoNi-MOF 50 μg mL^−1^) for 30 min and then dried at room temperature for 10 min. A drop of TP solution (10 mM, 2 μL) was added to the paper strip to test the detection ability of the paper strip sensor.

To explore the LOD of the paper strip sensor, 2 μL of TP solutions with different concentrations (0.02 mM to 10 mM) was dropped on the paper strips. Additionally, the interference of interfering substances (Fe^3+^, NH_4_^+^, Ni^2+^, HCO_3_^−^, PO_4_^3−^, Ac^−^, PFOA, P, 2, 4-D, 2, 4-CP) with the concentration of 0.5 mM and the time of the paper strip sensor remaining active were also investigated. The activity of the paper strip sensor was evaluated by observing the color with the naked eye.

The reagents and instruments required in the experiments, specific experimental details of optimization, steady-state kinetic analysis, exploration of detection principles of TP, and the selectivity of the colorimetric sensor as well as sample collection and processing are shown in the Supporting Information.

## 4. Conclusions

In summary, we report a highly specific trimetallic nanozyme-like material FeCoNi-MOF by partially replacing the original Fe nodes of the Fe-MOF with Co and Ni nodes. Compared with monometallic/bimetallic MOFs containing equimolar metals, FeCoNi-MOF exhibits much higher peroxidase-like activity, benefiting from the synergistic electron transfer effect between Fe, Co and Ni. Furthermore, employing FeCoNi-MOF works as an accurate, stable and sensitive unit for colorimetric sensor construction; faster reaction speed (~14.30 × 10^−8^ M s^−1^) and stronger affinity with TMB (K_m_ = 0.061 mM) as well as H_2_O_2_ (K_m_ = 0.074 mM) are obtained. Two sensing platforms based on FeCoNi-MOF have been constructed successfully for the colorimetric detection of H_2_O_2_ and TP with lower LODs (H_2_O_2_: 1.75 μM; TP: 0.045 μM) and wider linear ranges (H_2_O_2_: 6–800 μM; TP: 0.5–80 μM). Finally, the newly built TP sensing platform has been successfully applied to TP sensing in four real water samples with average recoveries ranging from 94.6% to 112.1%. This work is an eager attempt to achieve the design, construction and application of polymetallic nanozymes. In addition, benefiting from its good dual-enzyme mimetic catalytic activities, high efficiency and excellent stability as well as ease of preparation, FeCoNi-MOF is expected to find applications in biosensing, pollutant detection and degradation, biological imaging, immunoassays and therapies.

## Figures and Tables

**Table 1:** Comparison of K_m_ values and V_max_ values with different MOFs and H_2_O_2_ as the fixed substrate.

	V_max_ (10^−8^ M s^−1^)	K_m_ (mM)	Double Reciprocal Equation	R^2^
Fe-MOF	5.32	0.318	y = 5977.44x + 18.80	0.999
FeCo-MOF	10.28	0.135	y = 1313.23x + 9.73	0.994
FeNi-MOF	9.17	0.198	y = 2159.22x + 10.91	0.996
CoNi-MOF	8.34	0.251	y = 3009.59x + 11.99	0.990
FeCoNi-MOF	13.68	0.054	y = 392.44 + 7.31	0.997

**Table 2:** Comparison of K_m_ values and V_max_ values with different MOFs and TMB as the fixed substrate.

	V_max_ (10^−8^ M s^−1^)	K_m_ (mM)	Double Reciprocal Equation	R^2^
Fe-MOF	5.98	0.323	y = 5401.34x + 16.72	0.999
FeCo-MOF	11.42	0.142	y = 1243.43x + 8.76	0.998
FeNi-MOF	10.31	0.188	y = 1823.47x + 9.70	0.996
CoNi-MOF	9.26	0.237	y = 2559.40x + 10.80	0.995
FeCoNi-MOF	14.43	0.082	y = 566.85x + 6.93	0.997

**Table 3:** K_m_ values, V_max_ values and the average values of them obtained under different substrate concentrations.

	The Concentration of the Fixed Substrate (μM)	V_max_ (10^−8^ M s^−1^)	K_m_ (mM)
Fix the concentration of H_2_O_2_ and change the concentration of TMB	50	11.89	0.052
	100	13.93	0.071
	200	13.68	0.054
	500	13.79	0.068
	Average	13.32	0.061
Fix the concentration of TMB and change the concentration of H_2_O_2_	100	7.87	0.053
	200	14.43	0.082
	500	16.37	0.102
	1000	18.52	0.059
	Average	14.30	0.074

**Table 4:** Analytical results for the determination of the TP in four actual water samples.

	Added TP (μM)	Average Detected TP (μM)	Average Recovery (%)	RSD (%)
Tap water	0	0	-	6.47
	2	1.994	99.7	1.89
	5	5.106	102.1	3.78
	10	10.874	108.7	0.43
	20	21.183	105.9	0.22
Jiuxiang River water	0	0	-	5.83
	2	1.891	94.6	7.32
	5	5.078	101.6	4.56
	10	10.258	102.6	0.87
	20	19.912	99.6	0.11
Xuanwu Lake water	0	0	-	0.57
	2	2.108	105.4	2.12
	5	4.992	99.8	3.44
	10	11.091	110.9	4.02
	20	20.453	102.3	2.55
Tai Lake water	0	0	-	4.11
	2	2.102	105.1	5.63
	5	5.117	102.3	0.91
	10	11.213	112.1	5.59
	20	19.576	97.9	6.02

## Data Availability

The original contributions presented in the study are included in the article/Supplementary Material, further inquiries can be directed to the corresponding author.

## Supplementary Materials

The following supporting information can be downloaded at: https://www.mdpi.com/article/10.3390/molecules29163739/s1. Scheme S1. Mechanism showing catalytic capacity for the activation of H_2_O_2_ of CoNi-MOF and FeCoNi-MOF. Figure S1. Optical photos of different MOF materials. Figure S2. Elemental mapping images for C, Fe, O of Fe-MOF. Figure S3. Elemental mapping images for C, Co, O of Co-MOF. Figure S4. Elemental mapping images for C, Ni, O of Ni-MOF. Figure S5. Elemental mapping images for C, Fe, Co, O of FeCo-MOF. Figure S6. Elemental mapping images for C, Fe, Ni, O of FeNi-MOF. Figure S7. Elemental mapping images for C, Co, Ni, O of CoNi-MOF. Figure S8. Elemental mapping images for C, Fe, Co, Ni, O of FeCoNi-MOF. Figure S9. Content and molar ratios of metal elements in MOF materials by SEM-EDS mapping. Figure S10. The N_2_ adsorption-desorption isotherms obtained at −196 °C of seven MOFs. Figure S11. Optimization of the experimental conditions which have a great effect on the color effect of the nanozyme sensor: (A) Influence of the concentration of FeCoNi-MOF; (B) Influence of the pH of the system; (C) Influence of the concentration of TMB; (D) Influence of the reaction time; (E) Influence of the temperature. (The absorbance of TMB at 652 nm is the basis for quantification). Figure S12. The H_2_O_2_ quantification results using constructed nanozyme colorimetric sensor. (A) The UV absorption spectra of different systems with different concentrations of H_2_O_2_ added (0–10,000 μM). (B) Corresponding optical photograph of reaction systems with different concentrations of H_2_O_2_ (0–6000 μM); (C) The scatter plot (absorbance at 652 nm) for H_2_O_2_ detection using nanozyme sensor with FeCoNi-MOF as the catalyst in the range of 0–10,000 μM; (D) The linear calibration plot (absorbance at 652 nm) for TP detection using nanozyme sensor with FeCoNi-MOF as the catalyst in the range of 6–800 μM. Figure S13. Variation of the absorbance at 652 nm showing sensing ability of fabricated nanozyme sensor toward H_2_O_2_ and various interferences (300 μM). Inset: Optical photographs showing sensing ability of fabricated nanozyme sensor toward various interferences (The numerical labels represent the pollutant types marked from left to right on the abscissa, respectively). Conditions: FeCoNi-MOF (25 μg/mL), TMB (0.5 mM) in acetate buffer (pH 4.0), kept at 40 °C for 40 min to reach equilibrium. Figure S14. Steady-state kinetic assay of FeCoNi-MOF as the nanozyme with H_2_O_2_ and TMB as the substrate: Michaelis-Menten curves of FeCoNi-MOF with the concentration of H_2_O_2_ fixed and the concentration of TMB varied. Fix the concentration of H_2_O_2_ at (A) 50 μM; (B) 100 μM; (C) 200 μM; (D) 500 μM. Figure S15. Steady-state kinetic assay of FeCoNi-MOF as the nanozyme with H_2_O_2_ and TMB as the substrate: Double-reciprocal plots of FeCoNi-MOF with the concentration of H_2_O_2_ fixed and the concentration of TMB varied. Fix the concentration of H_2_O_2_ at (A) 50 μM; (B) 100 μM; (C) 200 μM; (D) 500 μM. Figure S16. Steady-state kinetic assay of FeCoNi-MOF as the nanozyme with H_2_O_2_ and TMB as the substrate: Michaelis-Menten curves of FeCoNi-MOF with the concentration of TMB fixed and the concentration of H_2_O_2_ varied. Fix the concentration of TMB at (A) 100 μM; (B) 200 μM; (C) 500 μM; (D) 1000 μM. Figure S17. Steady-state kinetic assay of FeCoNi-MOF as the nanozyme with H_2_O_2_ and TMB as the substrate: Double-reciprocal plots of FeCoNi-MOF with the concentration of TMB fixed and the concentration of H_2_O_2_ varied. Fix the concentration of TMB at (A) 100 μM; (B) 200 μM; (C) 500 μM; (D) 1000 μM. Figure S18. Double-reciprocal plots of activity of FeCoNi-MOF at a fixed concentration of one substrate versus varying concentration of the second substrate for H_2_O_2_ and TMB. (A) Fix the concentration of H_2_O_2_ and vary the concentration of TMB; and (B) fix the concentration of TMB and vary the concentration of H_2_O_2_. Figure S19. The catalytic performance of FeCoNi MOF after reuse. Reaction condition: 2 mg mL^−1^ FeCoNi MOF, 500 μM TMB, 500 μM H_2_O_2_, pH 4.0, 20 min, under 40 oC in 5 mL reaction system. Figure S20. Absorbance of different systems at 652 nm (suspension of FeCoNi-MOF 25 μg mL^−1^, TP 50 μΜ, TMB 500 μΜ and H_2_O_2_ 400 μΜ in pH 4.0 acetate buffer (0.1 M), the total volume 1 mL). Blank: Without added TP. System 1: TP was added firstly and then TMB, H_2_O_2_ and suspension of FeCoNi-MOF were added in sequence. Next, the system was incubated at 40 °C for 20 min. System 2: TMB, H_2_O_2_ and suspension of FeCoNi-MOF were added in sequence and the system was incubated at 40 °C for 20 min similarly. Finally, TP was added and the system continued to incubate at 40 °C for 20 min. Figure S21. EPR spectra in the activation process of H_2_O_2_ under different conditions. Figure S22. (A) Three different forms of TMB in acidic pH and (B) mechanism of the oxidation of TMB to oxTMB by free radicals and the restoration of oxTMB to TMB by the hydrogen donation of TP. Figure S23. Variation of the absorbance at 652 nm showing sensing ability of fabricated nanozyme-like sensors toward TP and various interferences (50 μM): (A) Common anions and cations; and (B) Common organic pollutants. Inset: Optical photographs showing sensing ability of fabricated nanozyme sensor toward various interferences (The numerical labels represent the pollutant types marked from left to right on the abscissa, respectively). Conditions: FeCoNi-MOF (25 μg mL^−1^), TMB (0.5 mM) and H_2_O_2_ (0.4 mM) in acetate buffer (pH 4.0), kept at 40 °C for 40 min to reach equilibrium. Figure S24. Variation of the absorbance at 652 nm of the sensing system incubated with TP and various interferences (TP: 50 μM and other interferences: 100 μM). Conditions: FeCoNi-MOF (25 μg mL^−1^), TMB (0.5 mM) and H_2_O_2_ (0.4 mM) in acetate buffer (pH 4.0), kept at 40 °C for 40 min to reach equilibrium. Figure S25. The UV absorption spectra of different reaction systems of actual water samples spiked with different concentrations of TP (0 μM, 2 μM, 5 μM, 10 μM and 20 μM): (A) Tap water; (B) Jiuxiang River Water; (C) Xuanwu Lake water; (D) Tai Lake water. Inset: Corresponding optical photographs of different reaction systems of actual water samples. Conditions: FeCoNi-MOF (25 μg mL^−1^), TMB (0.5 mM), H_2_O_2_ (0.4 mM) in acetate buffer (pH 4.0), kept at 40 °C for 40 min to reach equilibrium. Figure S26. (A) Activity of paper trip sensor at varying fabrication time (min) (using 2 µL, TP conc., 0.5 mM); (B) Activity of paper sensor in presence of mixture of other pollutants (Fe^3+^, NH^4+^, Ni^2+^, HCO^3−^, PO_4_^3−^, Ac^−^, PFOA, P, 2, 4-D, 2, 4-CP, 0.5 mM): (1) 2 µL of pure TP solution (0.5 mM); (2) 2 µL of the mixture of TP and other pollutants (containing TP and each pollutant of 0.5 mM). (3) 2 µL of the mixture of other pollutants (containing each pollutant of 0.5 mM); (4) Standard solution without TP and other pollutants. Figure S27. Analysis of the paper strip sensor by ImageJ_v1.8.0. Table S1. The peroxidase-like and oxidase-like activities of seven MOFs. Table S2. Method comparisons for the analysis of the H_2_O_2_. Table S3. Comparison of K_m_ values and V_max_ values with TMB as the substrate. Table S4. Comparison of K_m_ values and V_max_ values with H_2_O_2_ as the substrate. Table S5. Method comparisons for the analysis of the TP. Table S6. Analytical data of the sensor. Table S7. Properties of the distilled water and actual water samples. Table S8. The grayscale quantization values of three paper strip sensors. Refs. [66,67,68,69,70,71,72,73,74,75,76,77,78,79,80,81,82,83,84,85,86,87,88,89,90] are cited in the Supplementary Materials.
