# Supplementary material for: Trimetallic FeCoNi Metal–Organic Framework with Enhanced Peroxidase-like Activity for the Construction of a Colorimetric Sensor for Rapid Detection of Thiophenol in Water Samples
Source: Molecules. 2024 Aug 7;29(16):3739. doi: 10.3390/molecules29163739 (PMC11356859; doi:10.3390/molecules29163739)
Supplement: Supplementary file 1 [file molecules-29-03739-s001.zip › molecules-3096484-supplementary.pdf]

Supporting Information

Trimetallic FeCoNi Metal–Organic Framework with Enhanced Peroxidase-like Activity for the Construction of a Colorimetric Sensor for Rapid Detection of Thiophenol in Water Samples

Zehui Deng [1,2], Jiaqing Cao [3], Lei Zhao [3], Zhao Zhang [3] and Jianwei Yuan [3,4,*]

[1] State Key Laboratory of Pollution Control and Resource Reuse, School of Environment, Nanjing University, Nanjing 210023, China; dg1925004@smail.nju.edu.cn

[2] Shandong Institute of Metrology, Jinan 250014, China

[3] School of Chemical Engineering and Materials, Changzhou Institute of Technology, 666 Liaohe Road (S), Changzhou 213022, China; caojq@czust.edu.cn (J.C.); zhaol@czust.edu.cn (L.Z.); zhangz@czust.edu.cn (Z.Z.)

[4] State Key Laboratory of Materials-Oriented Chemical Engineering, College of Chemical Engineering, Nanjing Tech University, 30 Puzhu Road (S), Nanjing 211816, China

* Correspondence: yuanjw@czust.edu.cn

Table of Contents

Experimental Section………………………………………...…………….. S4

Chemicals and reagents…………...S4

Instruments and characterizations…………..S5

The synthesis processes of seven MOFs with different metal ions……………..... S6

Optimization of nanozyme-like colorimetric sensor we constructed……………..S7

Steady-state kinetic analysis of H_2O_2 activation on $Co(BDC)TED_{0.5}$…………………………………………………………………...S8

The selectivity of this colorimetric sensor…………..S10

Exploration of detection principles of TP…………..S10

Sample collection and processing…………...S11

Discussion Section………………………………………..…...……..... S12

Optimization of nanozyme colorimetric sensor we constructed…...……...……...S12

The sensitivity of H_2O_2 detection…………..S14

The changes of the form of TMB in the whole detection process…...……...……...S15

The selectivity of the nanozyme sensor…………..S16

Supplementary Scheme……………………………..………………..… S17

Scheme S1……………………………………………………………………..S17

Supplementary Figures……………………………………..…………... S18

Fig. S1…………………………………………………………………..…… S18

Fig. S2…………………………………………………………………..…… S19

Fig. S3…………………………………………………………………..…… S19

Fig. S4…………………………………………………………………..……. S20

Fig. S5……………………………………………………………………..... S20

Fig. S6…………………………………………………………………….... **S21**

Fig. S7……………………………………………………………..……… **S22**

Fig. S8……………………………………………………………...…… **S23**

Fig. S9……………………………………………………………...…… **S23**

Fig. S10……………………………………………………………...... **S24**

Fig. S11……………………………………………………………...... **S25**

Fig. S12……………………………………………………………...... **S26**

Fig. S13……………………………………………………………...... **S27**

Fig. S14……………………………………………………………...... **S28**

Fig. S15……………………………………………………………...... **S29**

Fig. S16……………………………………………………………...... **S30**

Fig. S17……………………………………………………………...... **S31**

Fig. S18……………………………………………………………...... **S32**

Fig. S19……………………………………………………………...... **S33**

Fig. S20……………………………………………………………...... **S34**

Fig. S21……………………………………………………………...... **S35**

Fig. S22…………………………………………………………............ **S36**

Fig. S23…………………………………………………………............ **S37**

Fig. S24…………………………………………………………............ **S38**

Fig. S25…………………………………………………………............ **S39**

Fig. S26…………………………………………………………............ **S40**

Fig. S27…………………………………………………………............ **S41**

Supplementary Tables………………………………………………….... **S42**

Table S1…………………………………………………………..…..... **S42**

Table S2…………………………………………………………..…..... **S43**

Table S3………………………………………………………………………..….. S44

Table S4………………………………………………………………………..….. S46

Table S5………………………………………………………………………..….. S48

Table S6………………………………………………………………………..….. S49

Table S7………………………………………………………………………..….. S50

Table S8………………………………………………………………………..….. S51

References………………………………………………………………………. S52

Experimental Section

Chemicals and reagents

Cobalt (II) chloride hexahydrate ($CoCl_2 \cdot 6H_2O$) (99%), nickel (II) chloride hexahydrate ($NiCl_2 \cdot 6H_2O$) (99%), iron (II) chloride tetrahydrate ($FeCl_2 \cdot 4H_2O$) (99%), ferric (III) chloride hexahydrate ($FeCl_3 \cdot 6H_2O$) (99%), terephthalic acid (H_2BDC) (98%), pentadecafluorooctanoic acid (PFOA) (98%), bisphenol A (BPA) (>99.8%), 4-nonylphenol (4-NP) (98%), phenol (P) (≥99.5%), 2,4-dichlorophenol (2,4-CP) (≥99.5%) and sulfadiazine (SD) (98%) were obtained from Shanghai Aladdin Biochemical Technology Co. Ltd. (Shanghai, China, www.aladdin-e.com). Perfluorooctanesulfonic acid (PFOS) (98%), 2,4-dichlorophenoxyacetic acid (2, 4-D) (98%) and 4-octylphenol (4-OP) (99%) were obtained from J&K Scientific Co. Ltd. (Beijing, China, www.jkchemical.com). Thiophenol (TP, ≥99%), 2, 2, 6, 6-tetramethylpiperidine (TEMP) (≥99%) and 5,5-dimethyl-1-pyrroline-N-oxide (DMPO) were purchased from Sigma-Aldrich Shanghai Trading Co., Ltd. (www.sigmaaldrich.com/china-mainland.html). 3,3',5,5'-Tetramethylbenzidine (TMB) (BR) was purchased from Shanghai D&B Biological Science and Technology Co. Ltd (Shanghai, China, www.chemxyz.com). Hydrogen peroxide (H_2O_2) (≥30%), hydrofluoric acid (HF) (≥40%), sodium acetate anhydrous (CH_3COONa), acetic acid (CH_3COOH), N, N-dimethylformamide (DMF), tert-butyl alcohol, sodium chloride (NaCl), potassium chloride (KCl), sodium bromide (NaBr), ammonium chloride (NH_4Cl), sodium bicarbonate ($NaHCO_3$), disodium hydrogen phosphate dodecahydrate ($Na_2HPO_4 \cdot 12H_2O$), potassium dihydrogen phosphate (KH_2PO_4), sodium phosphate

tribasic dodecahydrate ($Na_3PO_4 \cdot 12H_2O$) and ethanol were provided by Sinopharma Chemical Reagent Co. Ltd. (Shanghai, China, www.sinopharmholding.com). Methanol was purchased from Shanghai Titan Scientific Co. Ltd. (Shanghai, China, www.tansoole.com). L-Alanine (Ala), L-arginine (Arg), L-proline (Pro), L-histidine (His), glucose and sucrose were purchased from Shanghai Yuanye Biological Technology Co. Ltd. (Shanghai, China, www.shyuanye.com/). Without special instructions, the reagents are of analytical grade and are used as received without further purification.

Instruments and characterizations

Photomicrograph images of the four materials were obtained by FESEM (Zeiss SUPPA™ 55, Germany). XRD measurements were conducted with Cu Kα radiation on a D/max-Rb diffractometer (Rigaku, Japan) over an angular range from 5° to 80°. FT-IR spectra were collected using the KBr pellet technique on the Nicolet iS 10 infrared spectrometer (Thermo Scientific, Waltham, MA, USA). Roman spectra were collected using environmental X-ray Raman spectroscopy (XPS, XploRA™ PLUS, HOROBA). X-ray photoelectron spectroscopy (XPS) was performed on a PHI-5000 Versa Probe using a mono-chromatized Al Kα excitation source (hν=1486.6 eV) (ULVACPHI, Japan). The C1s peak (284.8 eV) was used for the calibration of binding energy. The absorbance at 652 nm was measured using a UV−vis spectrophotometer (model 2450, Shimadzu Co., Japan). The pH values of the reaction systems were measured using the pH meter (model FE20, Mettler-Toledo Co., Germany). Free radicals were detected on an electron paramagnetic resonance (EPR) spectrometer (EMXplus, Bruker Co.,

Germany).

The synthesis processes of seven MOFs with different metal ions

Seven MOFs with different metal ions were synthesized according to the reference with some modifications **[1, 2]**.

Fe-MOF: 299 mg (1.5 mmol) $FeCl_2·4H_2O$ and 249 mg (1.5 mmol) H_2BDC were dissolved in the mixed solution of H_2O (2.5 mL), ethanol (2.5 mL) and DMF (35 mL), and then 0.15 mL HF was added into mixture. The mixture was transferred into 100 ml Teflon-lined autoclave and heated at 150 °C for 15 h.

Co-MOF: 357 mg (1.5 mmol) $CoCl_2·6H_2O$ and 249 mg (1.5 mmol) H_2BDC were dissolved in the mixed solution of H_2O (2.5 mL), ethanol (2.5 mL) and DMF (35 mL), and then 0.15 mL HF was added into mixture. The mixture was transferred into 100 ml Teflon-lined autoclave and heated at 150 °C for 15 h.

Ni-MOF: 357 mg (1.5 mmol) $NiCl_2·6H_2O$ and 249 mg (1.5 mmol) H_2BDC were dissolved in the mixed solution of H_2O (2.5 mL), ethanol (2.5 mL) and DMF (35 mL), and then 0.15 mL HF was added into mixture. The mixture was transferred into 100 ml Teflon-lined autoclave and heated at 150 °C for 15 h.

FeCo-MOF: 149 mg (0.75 mmol) $FeCl_2·4H_2O$, 179 mg (0.75 mmol) $CoCl_2·6H_2O$ and 249 mg (1.5 mmol) H_2BDC were dissolved in the mixed solution of H_2O (2.5 mL), ethanol (2.5 mL) and DMF (35 mL), and then 0.15 mL HF was added into mixture. The mixture was transferred into 100 ml Teflon-lined autoclave and heated at 150 °C for 15 h.

FeNi-MOF: 149 mg (0.75 mmol) $FeCl_2·4H_2O$, 179 mg (0.75 mmol) $NiCl_2·6H_2O$

and 249 mg (1.5 mmol) H_2BDC were dissolved in the mixed solution of H_2O (2.5 mL), ethanol (2.5 mL) and DMF (35 mL), and then 0.15 mL HF was added into mixture. The mixture was transferred into 100 ml Teflon-lined autoclave and heated at 150 °C for 15 h.

CoNi-MOF: 179 mg (0.75 mmol) $CoCl_2 \cdot 6H_2O$, 179 mg (0.75 mmol) $NiCl_2 \cdot 6H_2O$ and 249 mg (1.5 mmol) H_2BDC were dissolved in the mixed solution of H_2O (2.5 mL), ethanol (2.5 mL) and DMF (35 mL), and then 0.15 mL HF was added into mixture. The mixture was transferred into 100 ml Teflon-lined autoclave and heated at 150 °C for 15 h.

FeCoNi-MOF: 99 mg (0.5 mmol) $FeCl_2 \cdot 4H_2O$, 119 mg (0.5 mmol) $CoCl_2 \cdot 6H_2O$, 119 mg (0.5 mmol) $NiCl_2 \cdot 6H_2O$ and 249 mg (1.5 mmol) H_2BDC were dissolved in the mixed solution of H_2O (2.5 mL), ethanol (2.5 mL) and DMF (35 mL), and then 0.15 mL HF was added into mixture. The mixture was transferred into 100 ml Teflon-lined autoclave and heated at 150 °C for 15 h.

Seven MOFs are washed and dried with the same way: When the autoclave was cooled to room temperature, the crystalline powder was collected via centrifugation and washed with ultra-pure water for three times and dry ethanol for three times. And then the materials were dried under vacuum at 60 °C for 12 h for further characterization and catalytic reaction.

Optimization of nanozyme-like colorimetric sensor we constructed

In order to determine the effects of pH and temperature, the system containing materials, TMB and H_2O_2(materials: 25 μg mL^{-1}, TMB: 0.5 mM, H_2O_2: 0.5 mM, system

volume: 1 mL, reaction time: 30 min) was stabilized at temperatures varying from 20 ºC to 70 ºC or it was constructed using buffers with pH of 3.0-9.0. The effects of the amount of TMB or the material were evaluated by adding 50 μL TMB solution with the concentration varying from 2 mM to 20 mM or 50 μL suspension of the material suspension of the material with the concentration varying from 0.1 mg mL-1 to 0.8 mg mL^{-1} to the reaction system (the most suitable temperature and pH value after optimization, H_2O_2: 0.5 mM, system volume: 1 mL, reaction time: 30 min). The most adequate reaction time was chosen by recording the absorbance of the mixture in the time range of 0-120 minutes with the spectrophotometer under time scan mode. The effect of H_2O_2 dosage was evaluated by adding 50 μL H_2O_2 solution with the concentration ranging from 0 to 500 μM. Theoretically, as the concentration of H_2O_2 increases, the yield of active species will increase, and the absorbance of the system at 652 nm will increase. Therefore, by measuring the absorbance of the system at 652 nm, the quantitative detection of H_2O_2 can be achieved at the same time. For the selectivity evaluation of H_2O_2 detection, 300 μM Na^+, K^+, glucose, sucrose, alanine (Ala), histidine (His), arginine (Arg), lysine (Lys) and proline (Pro) were chosen as the interfering substances. They were added to the colorimetric system containing TMB and the material (FeCoNi-MOF: 25 μg mL^{-1}, TMB: 500 μM, the system pH: 4.0, reaction time: 20 min, reaction temperature 40 ºC, system volume: 1 mL) respectively and changes of the absorbance at 652 nm of the system were recorded.

Steady-state kinetic analysis of H_2O_2 activation on FeCoNi MOF

For an enzymatic reaction, V_{max} is used to indicate the maximum rate of enzymatic

reaction and K_m is used to evaluate the affinity between the enzyme and the substrate. The values of the K_m and V_{max} are calculated by double reciprocal plot (see equation (2)). In principle, the smaller the K_m is, the stronger the affinity between the enzyme and the substrate is.

Michaelis-Menten equation was also used to describe the steady-state kinetic behaviors of nanozymes in some reports **[3-5]**. Hence, we speculated the steady-state kinetic processes of the catalytic reaction with seven MOFs can be fitted with Michaelis-Menten equation. The steady-state kinetics measurements with H_2O_2 and TMB as the substrates were used to verify our speculation. In the experiments, the concentration of one substrate is fixed and the concentration of the other varies, resulting in a curve of the relationship between substrate concentrations and initial enzymatic reaction velocities. The steady-state kinetic measurements of five representative MOFs (Fe-MOF, FeCo-MOF, FeNi-MOF, CoNi-MOF and FeCoNi-MOF) were conducted with 200 μM TMB or 200 μM H_2O_2 as the fixed substrate under the optimal conditions. (FeCoNi-MOF: 25 μg mL^{-1}, the system pH: 4.0, reaction temperature 40 °C, system volume: 1 mL). When TMB was as the varied substrate, the concentration of TMB varied from 50 μM to 1000 μM. And when H_2O_2 was as the varied substrate, the concentration of H_2O_2 varied from 50 μM to 1000 μM.)

Besides, further measurements of changing the concentrations of the fixed substrates (TMB: 0.1, 0.2, 0.5 and 1 mM; H_2O_2: 50, 100, 200 and 500 μM) for FeCoNi-MOF were carried out to study the reaction mechanism of nanozyme. The reaction conditions we adopt were the same as above, and the concentration of the varied

substrate varied within the range of 50-1000 μM.

The selectivity of this colorimetric sensor

Some common inorganic ions and molecular organic pollutants which exist in the natural water may interfere with the detection of TP. To determine the selectivity of our nanozyme sensor toward TP, the responses of TP and common inorganic ions (Fe^{3+}, NH_4^+, Ni^{2+}, Co^{2+}, HCO_3^-, PO_4^{3-}, Ac^-, Br^-) and molecular organic pollutants (BPA, PFOA, PFOS, 2, 4-D, P, 4-OP, 4-NP, 2, 4-CP, SD) were studied by adding them with the concentration of 50 μM into the reaction system (FeCoNi-MOF: 25 μg mL^{-1}, TMB: 500 μM, H_2O_2: 400 μM, the system pH: 4.0, reaction time: 20 min, reaction temperature 40 °C, system volume: 1 mL), respectively. Besides, the responses of TP (50 μM) with the coexistence of these interfering substances (100 μM) were also tested and the reaction system we adopt was the same as above. The changes of the absorbance at 652 nm were recorded using UV-Vis.

Exploration of detection principles of TP

To explore the detection principles of TP, two different systems (suspension of FeCoNi-MOF 25 μg mL^{-1}, TP 50 μM, TMB 500 μM and H_2O_2 400 μM in pH 4.0 acetate buffer (0.1M), the total volume 1 mL) were designed.

In system 1, TP was added firstly and then TMB, H_2O_2 and suspension of FeCoNi -MOF were added in sequence. Next, the system was incubated at 40 °C for 20 min to achieve the thorough conversion of TMB to blue oxTMB. And in system 2, TMB, H_2O_2 and suspension of FeCoNi-MOF were added in sequence and the system was incubated at 40 °C for 20 min similarly. Finally, TP was added and the system continued to

incubate at 40 ºC for 20 min. Besides, EPR tests of different systems were also carried out to explore the mechanism of TP detection further.

Sample collection and processing

Tap water, Tai Lake water, Xuanwu Lake water and Jiuxiang River water were used as real water samples to verify the reliability of the nanozyme colorimetric sensor we constructed. Tap water sample was collected from Qianpansheng Building in Xianlin Campus of Nanjing University. Tai Lake water was collected from northeast of the Tai Lake. Xuanwu Lake water was collected from southeast of the Xuanwu Lake and Jiuxiang River water was collected from Jiuxiang River near the Xianlin Campus of Nanjing University. Four water samples were filtered with 0.45-μm micropore membranes before stored in brown glass bottles at 4 ºC for subsequent detection experiments using colorimetric sensor.

Discussion Section

Optimization of nanozyme colorimetric sensor we constructed

In order to make the sensor have better color effect and higher sensitivity, many key parameters, such as the concentrations of FeCoNi-MOF and TMB in the system, pH, temperature and reaction time are optimized. The system volume is 1 mL.

Firstly, the concentration of FeCoNi-MOF in the system is optimized, because for the enzymatic reaction, the amount of nanozyme is crucial, which determines the degree of activation of H_2O_2. 25 mg FeCoNi-MOF were dissolved in the 50 mL pH 4.0 acetate buffer to form a solution of 500 μg mL^{-1} according to reported by previous work **[6]**. Different concentrations of FeCoNi-MOF varying from 5 to 40 μg mL^{-1} were used to test the effect of the concentration of FeCoNi-MOF in the system. As is shown in **Fig. S11A**, when the concentration of FeCoNi-MOF increases from 5 μg mL^{-1} to 25 μg mL^{-1}, the absorbance increases from 0.2815 to 1.0595. When the concentration of FeCoNi-MOF continues to increase, the absorbance remains basically unchanged. In order to obtain the best catalytic efficiency and save the material, 25 μg mL^{-1} was selected as the concentration of FeCoNi-MOF in the system for the following experiments.

The concentration of TMB in the system should be optimized, because better color effect can be obtained only by adding proper amount of TMB. Different concentrations of TMB in the system (100-1000 μM) were used to test the effect of the concentration of TMB. As is shown in **Fig. S11B**, when the concentration of TMB varies from 100 μM to 500 μM, the absorbance increases from 0.3683 to 1.0632. However, when the concentration of TMB increases continuously, the absorbance remains constant at about

1.06. So, 500 μM was set as the concentration of TMB in the system for the following experiments.

pH is also an important parameter in the construction of the colorimetric sensor. pH not only determines the existence state of H_2O_2, GSH and TMB, but also affects the color effect of the sensor further. Acetate buffer, phosphate buffer and ammonia buffer with pH varying from 3.0 to 10.0 were used to test the effect of pH on the reaction system. As is shown in **Fig. S11C**, when pH changes from 3.0 to 4.0, the absorbance increases from 0.7970 to 1.0920, but when pH increases from 4.0 to 8.0, the color effect becomes worse with the absorbance decreasing from 1.0920 to 0.4212. And there is almost no color change in the reaction systems under the pH conditions of 9.0-10.0. The nanozyme sensor has relatively good color effect in the pH range of 3.0-6.0, which may be due to the fact that the chromogenic reagent TMB will achieve better color effect under acidic and neutral conditions. In addition, decomposition (hydrolysis) of H_2O_2 tends to occur rapidly at the pH higher than 5.0, leading to loss of its oxidation activity toward TMB. And, the first pKa of TMB is estimated as 4.2. From the concentration-pH diagram of TMB, it has been revealed that in pH 4.0 of sodium acetate buffer, TMB molecule exists in three forms (I, II and III) (**Fig. S22**). For oxidizing this molecule to the corresponding radical cation, it is necessary for the amino group to be deprotonated. Due to the necessity of deprotonation of amino groups, form III cannot participate in the oxidation process. One electron oxidation of forms I and II, results in removal of one electron from the free amino groups of these forms. Overall, the best activity of TMB molecules is achieved at pH=4. To obtain the best color effect, the

acetate buffer with pH 4.0 was used to construct the colorimetric sensor in the following experiments.

The reaction time and temperature were also optimized with a series of experiments (**Fig. S11D** and **Fig. S11E**). The reaction system can reach the maximum absorbance of 1.0648 within 20 minutes, and the absorbance remains constant within 120 minutes. At the same time, the reaction system can achieve great color effect and the absorbance is about 1.07 in the temperature range of 40-70 °C. Therefore, the nanozyme-like sensor we constructed has wider range of applications under different conditions. For the following experiments, 20 min and 40 °C were selected as the most suitable test time and temperature for the sensor.

As a result, the conditions we adopted for the construction of the sensor in the following experiments are summarized as follows: 25 μg mL^{-1} and 500 μM as the concentrations of FeCoNi-MOF and TMB in the system, respectively, 4.0 as the system pH, 40 min as the reaction time and 40 °C as the reaction temperature.

The sensitivity of H_2O_2 detection

The concentration of H_2O_2 in the system is also one of the factors that we need to be optimized in the construction of nanozyme colorimetric sensor. In the process of optimizing the concentration of H_2O_2, the quantification amount of H_2O_2 can also be determined. Varied H_2O_2 concentrations ranging from 0 μM to 10000 μM were tested. As is shown in **Fig. S12A** and **Fig. S12C**, as when the concentration of H_2O_2 increases, the absorbance at 652 nm of the reaction system gradually increases, which can also be reflected by the change of the color of the reaction system (**Fig. S12B**). Moreover, as

can be seen from **Fig. S12D**, a good linear relationship between absorbance and H_2O_2 concentrations from 6 μM to 800 μM is obtained. The regression equation is y = 0.001x+0.666 and the correlation coefficient (R^2) is 0.996 (where y and x represent absorbance at 652 nm and the concentration of H_2O_2 in the system, respectively). The LOD is calculated to be 1.75 μM according to the formula equation mentioned above (LOD = 3 (SB/k)) and the LOQ is calculated to be 5.83 μM according to the equation formula (LOQ =10 (SB/k)). Clearly, the newly-built colorimetric sensor based on FeCoNi-MOF for H_2O_2 detection has much lower LOD and wider linear range than other methods reported in literatures **[3, 7-15]** (**Table S2**), which illustrates suggests the great application potential of this sensor.

In addition, Na^+, K^+, glucose, sucrose, Ala, His, Arg, Lys and Pro as potential interfering substances were used to verify the selectivity of sensor for the H_2O_2 detection. Their presence did not affect the absorbance at 652 nm significantly (A-A_0≤0.05), which demonstrated that these substances did not interfere with the detection system and the sensor had good selectivity for the detection of H_2O_2 (**Fig. S13**).

The changes of the form of TMB in the whole detection process

TMB exists in three forms in the acetate buffer with pH=4.0 (I, II and III) (**Fig. S22A**) from the concentration-pH diagram of TMB **[16]**. For TMB to be oxidized to oxTMB, the deprotonation of its amino groups is essential. So, only form I and form II can participate in the oxidation process. As is shown in **Fig. S22B**, under the catalysis for H_2O_2 of the FeCoNi-MOF, TMB loses two electrons and becomes blue oxTMB. After the addition of GSH, GSH converts to GS·, accompanying with the restoration of

the blue oxTMB to original TMB. The restoration ability of GSH via hydrogen donation has been reported by many researchers **[16, 17]**. That is to say, GSH can reduce the oxTMB to the TMB (mainly II and III) by hydrogen donation.

The selectivity of the nanozyme sensor

Selectivity is an important factor to assess the practicality and sensitivity of a detection method. To test the selectivity of the sensor, common cations (Fe^{3+}, NH_4^+, Ni^{2+}, Co^{2+}), anions (HCO_3^-, PO_4^{3-}, Ac^-, Br^-) and organic pollutants (BPA, PFOA, PFOS, 2, 4-D, P, 4-OP, 4-NP, 2, 4-CP, SD) with the concentration of 50 μM were added into the reaction system, respectively and the changes of the absorbance at 652 nm were recorded. The results are displayed in **Fig. S23A-B** and only TP can cause significant changes in the color and the absorbance at 652 nm of the system ($A-A_0=0.6701$). As a result, common pollutants have little impact on the practical TP detection ($A-A_0 < 0.07$). Besides, when TP (50 μM) coexists with other pollutants at high concentrations (100 μM), the determination of it is not interfered (see **Fig. S24**). Hence, it can be concluded that the newly-built sensor with good selectivity and strong anti-interference ability is suitable for the detection of TP.

Supplementary Scheme

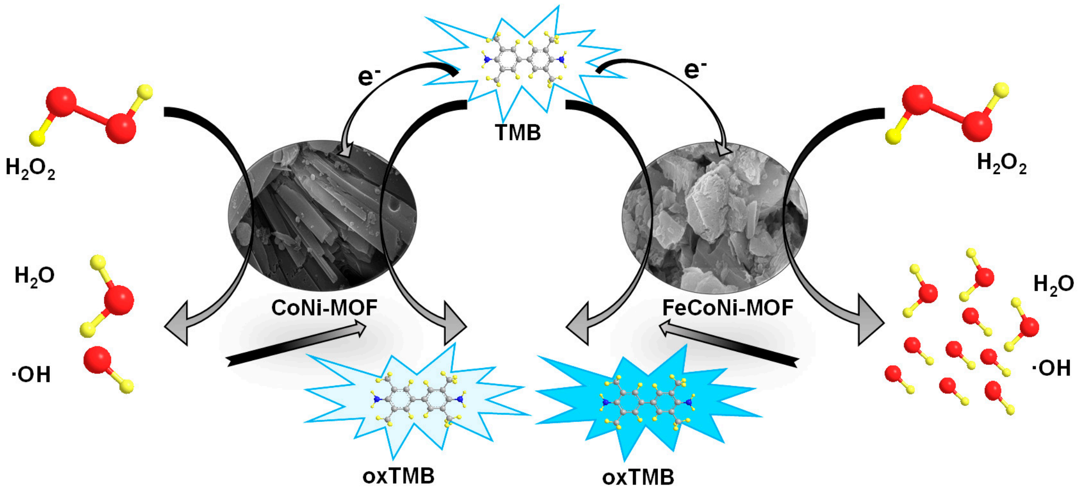

Scheme S1 Mechanism showing catalytic capacity for the activation of H_2O_2 of CoNi-MOF and FeCoNi-MOF.

Supplementary Figures

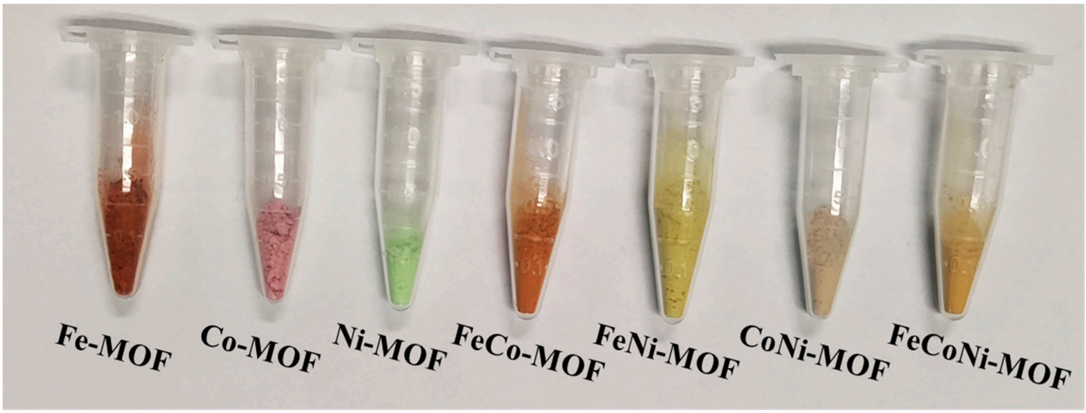

Fig. S1 Optical photos of different MOF materials.

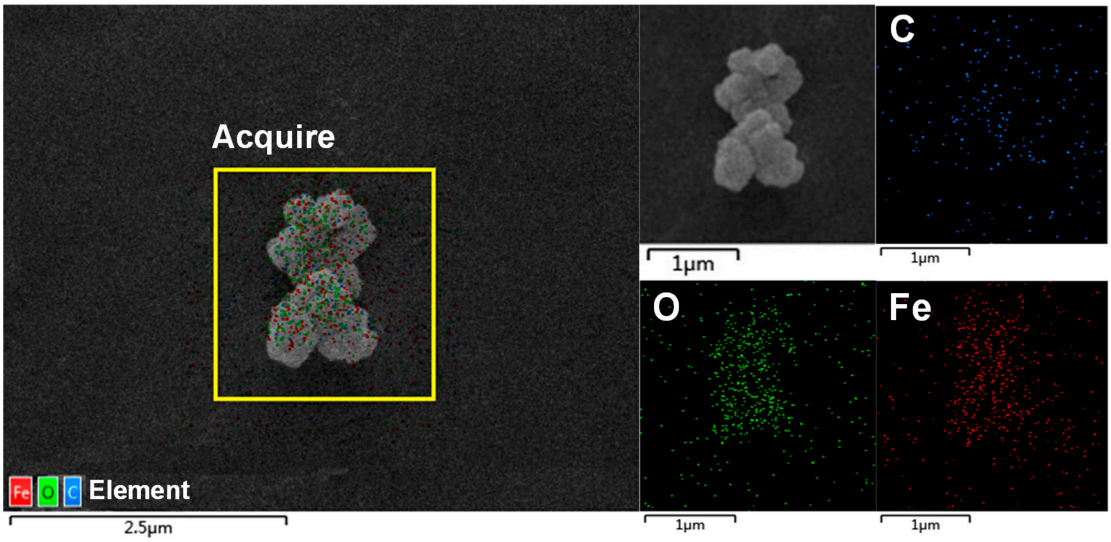

Fig. S2 Elemental mapping images for C, Fe, O of Fe-MOF.

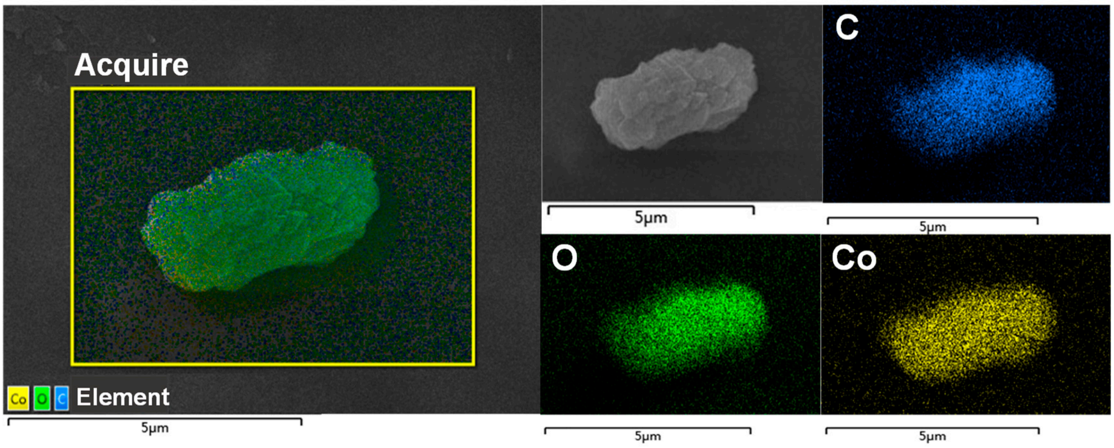

Fig. S3 Elemental mapping images for C, Co, O of Co-MOF.

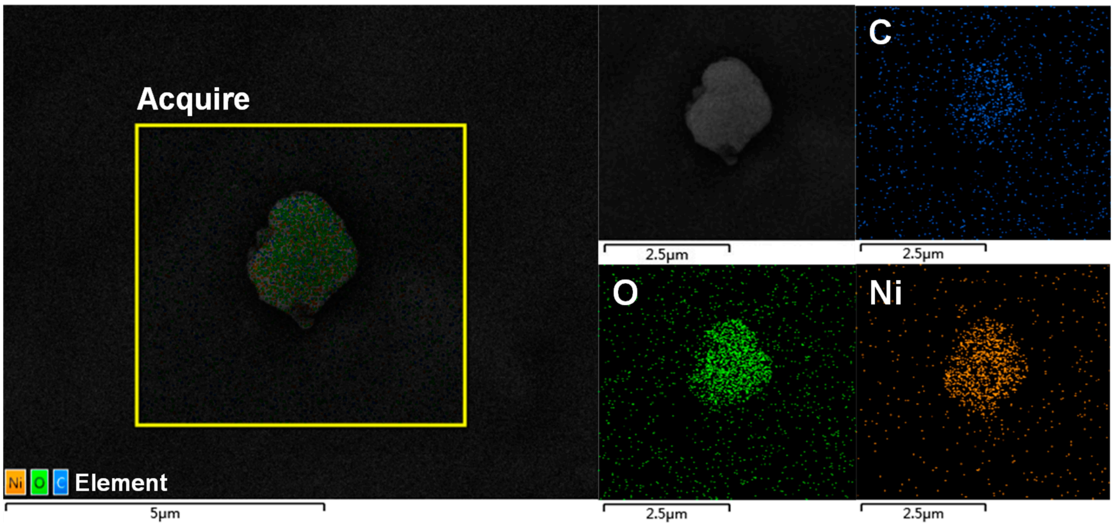

Fig. S4 Elemental mapping images for C, Ni, O of Ni-MOF.

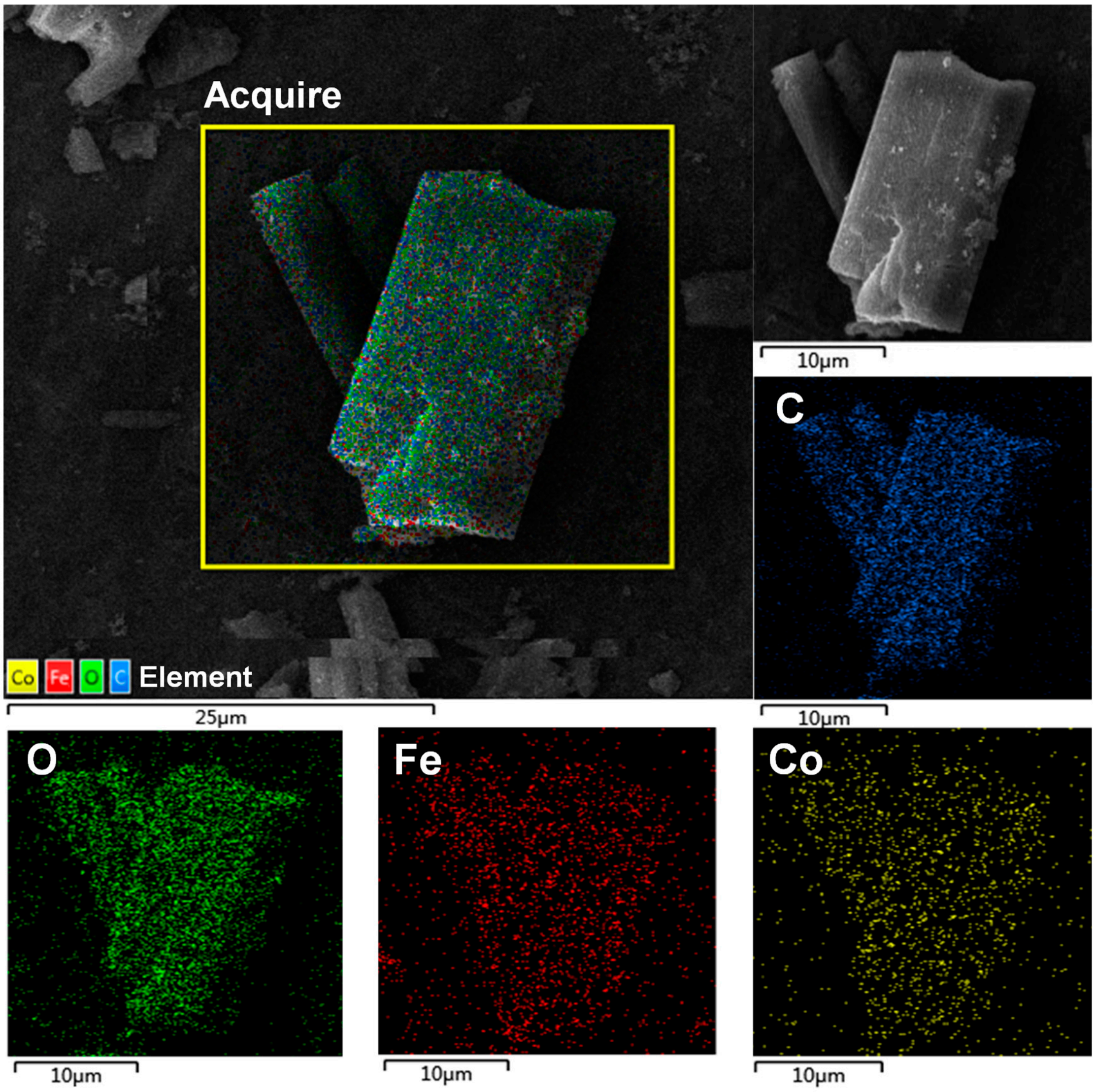

Fig. S5 Elemental mapping images for C, Fe, Co, O of FeCo-MOF.

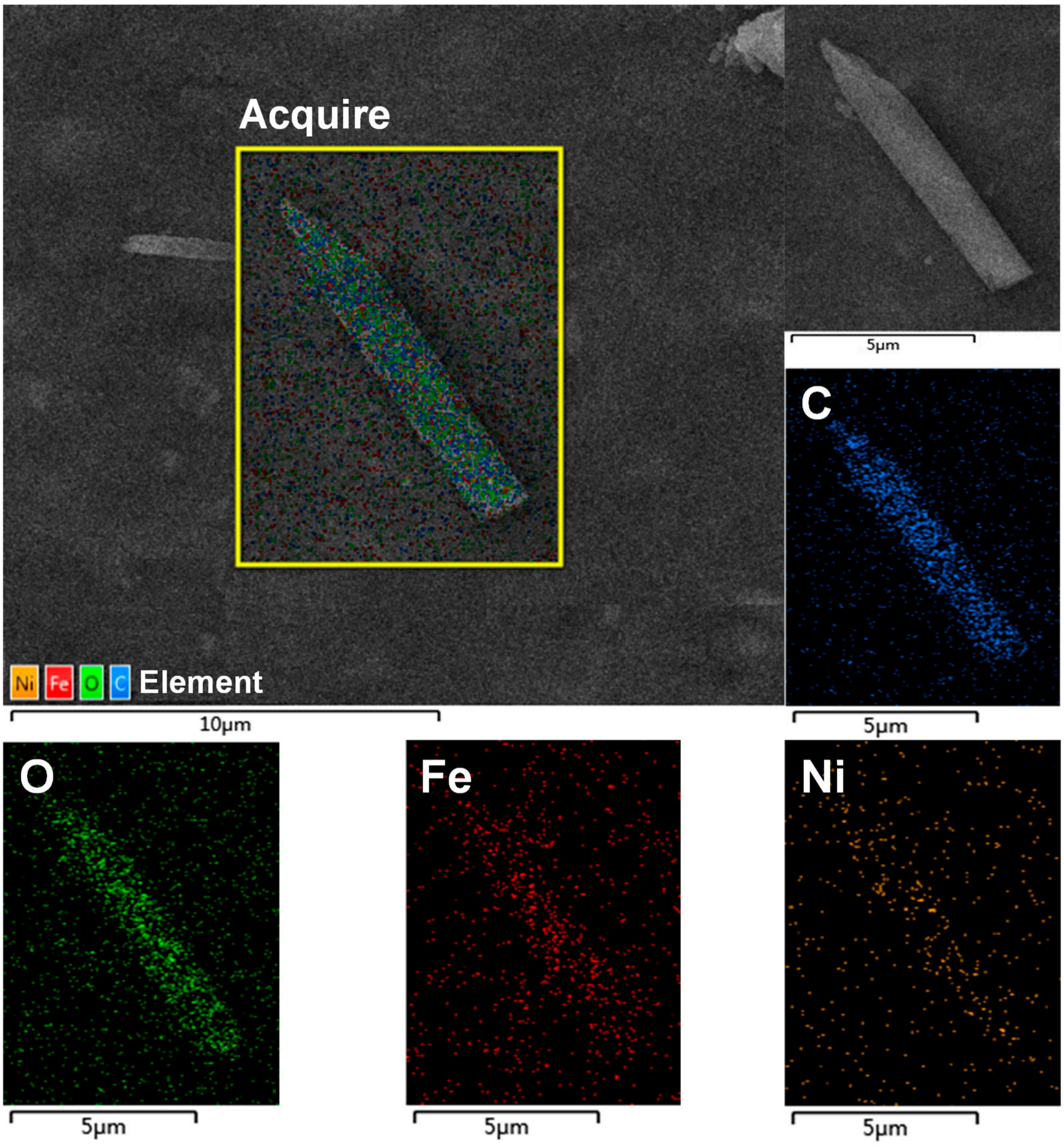

Fig. S6 Elemental mapping images for C, Fe, Ni, O of FeNi-MOF.

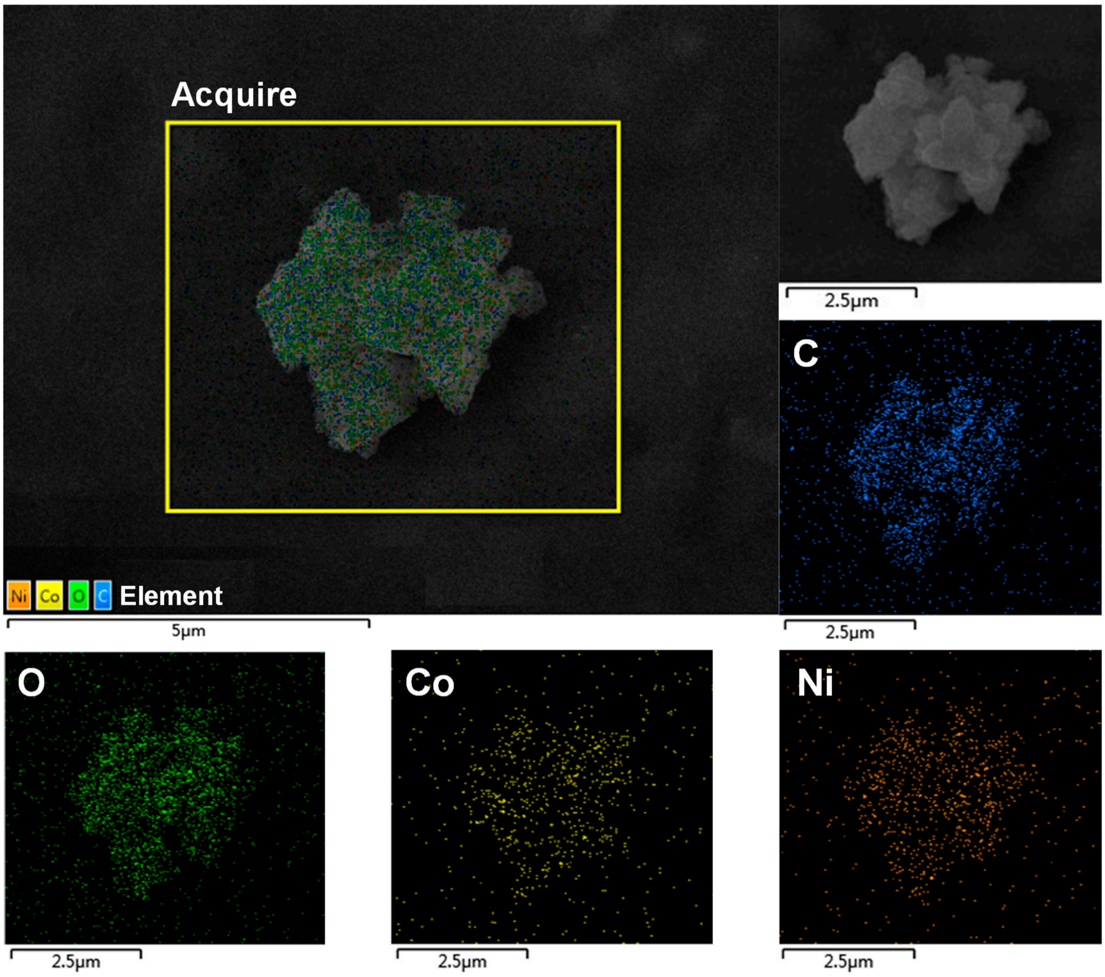

Fig. S7 Elemental mapping images for C, Co, Ni, O of CoNi-MOF.

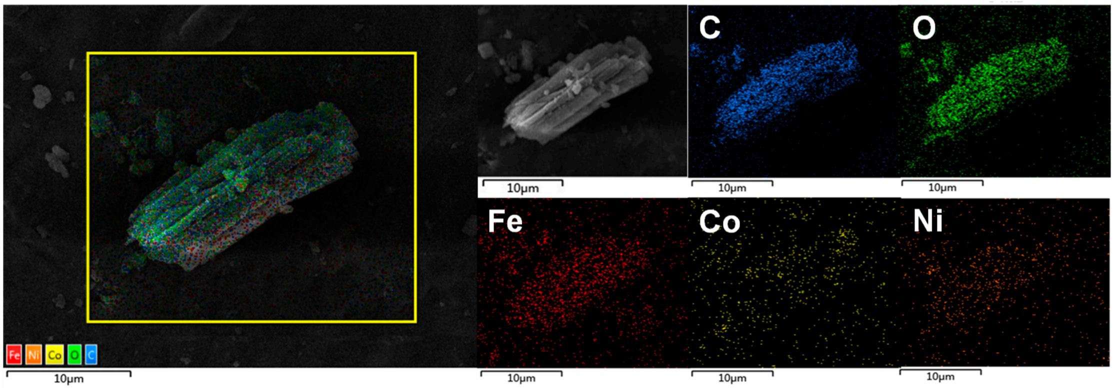

Fig. S8 Elemental mapping images for C, Fe, Co, Ni, O of FeCoNi-MOF.

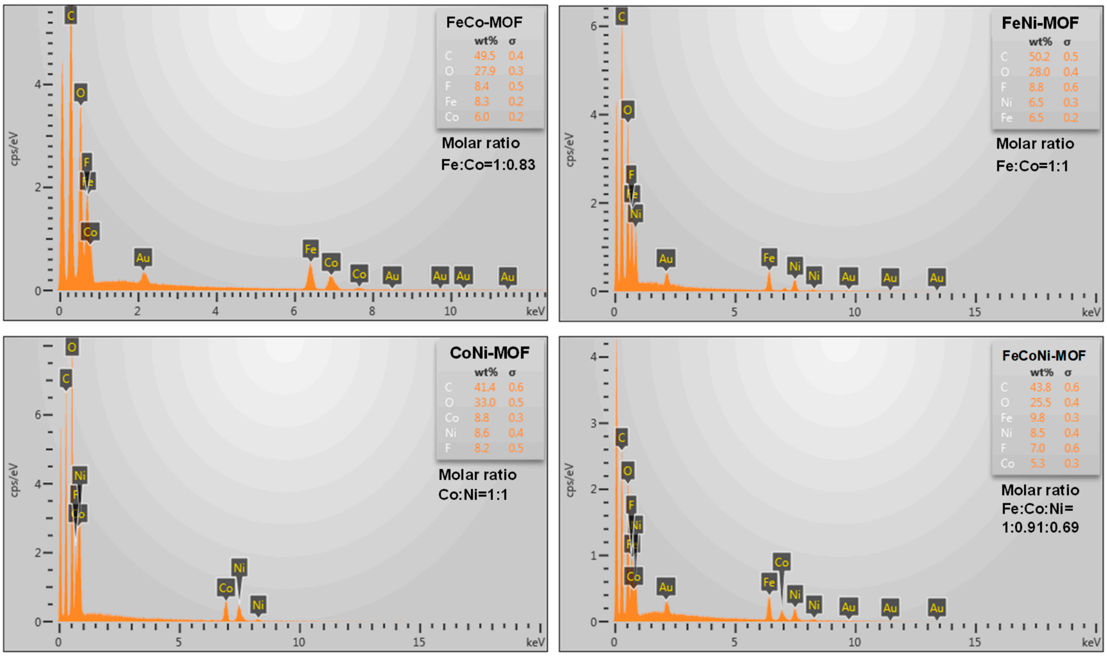

Fig. S9 Content and molar ratios of metal elements in MOF materials by SEM-EDS mapping.

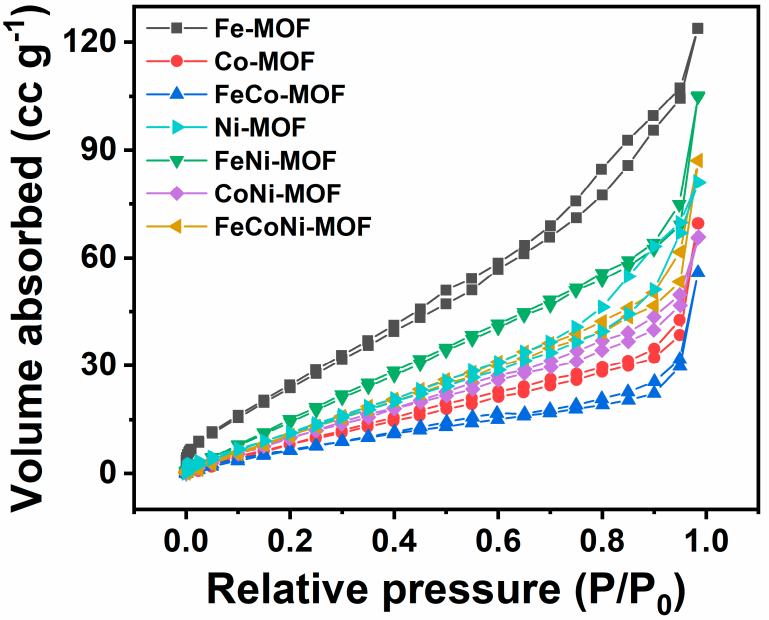

Fig. S10 The N_2 adsorption-desorption isotherms obtained at -196 ºC of seven MOFs.

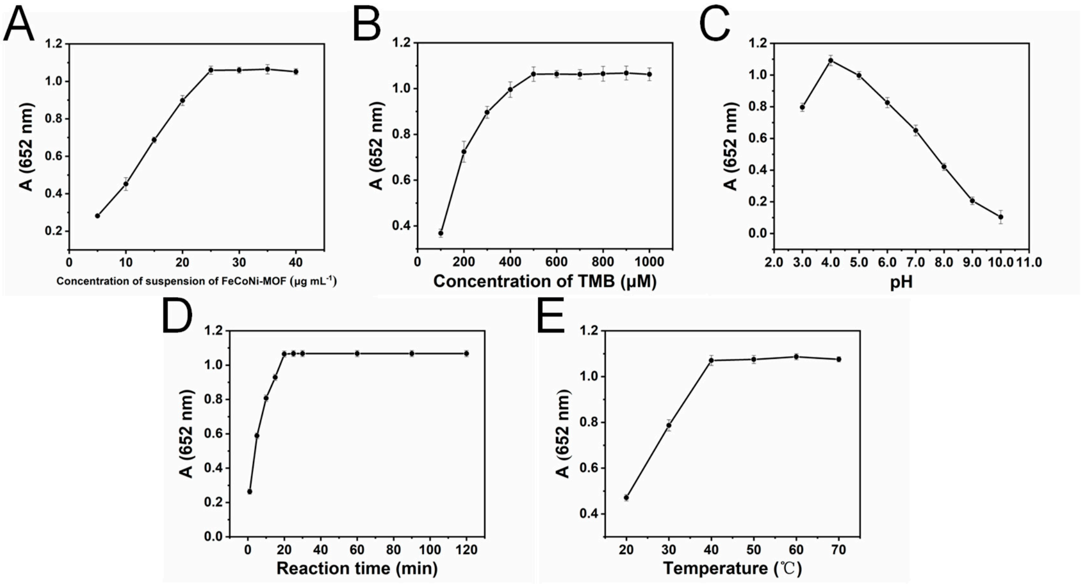

Fig. S11 Optimization of the experimental conditions which have a great effect on the color effect of the nanozyme sensor: (A) Influence of the concentration of FeCoNi-MOF; (B) Influence of the pH of the system; (C) Influence of the concentration of TMB; (D) Influence of the reaction time; (E) Influence of the temperature. (The absorbance of TMB at 652 nm is the basis for quantification.)

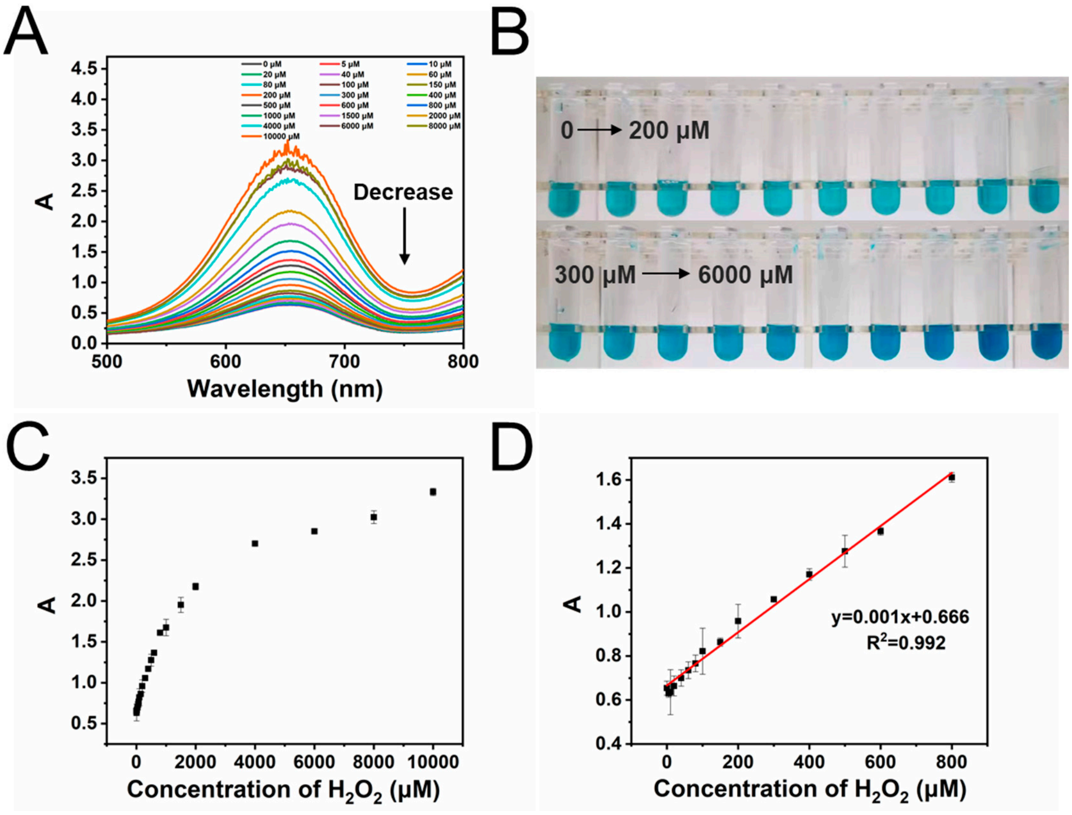

Fig. S12 The H_2O_2 quantification results using constructed nanozyme colorimetric sensor. (A) The UV absorption spectra of different systems with different concentrations of H_2O_2 added (0-10000 μM). (B) Corresponding optical photograph of reaction systems with different concentrations of H_2O_2 (0-6000 μM); (C) The scatter plot (absorbance at 652 nm) for H_2O_2 detection using nanozyme sensor with FeCoNi-MOF as the catalyst in the range of 0-10000 μM; (D) The linear calibration plot (absorbance at 652 nm) for TP detection using nanozyme sensor with FeCoNi-MOF as the catalyst in the range of 6-800 μM.

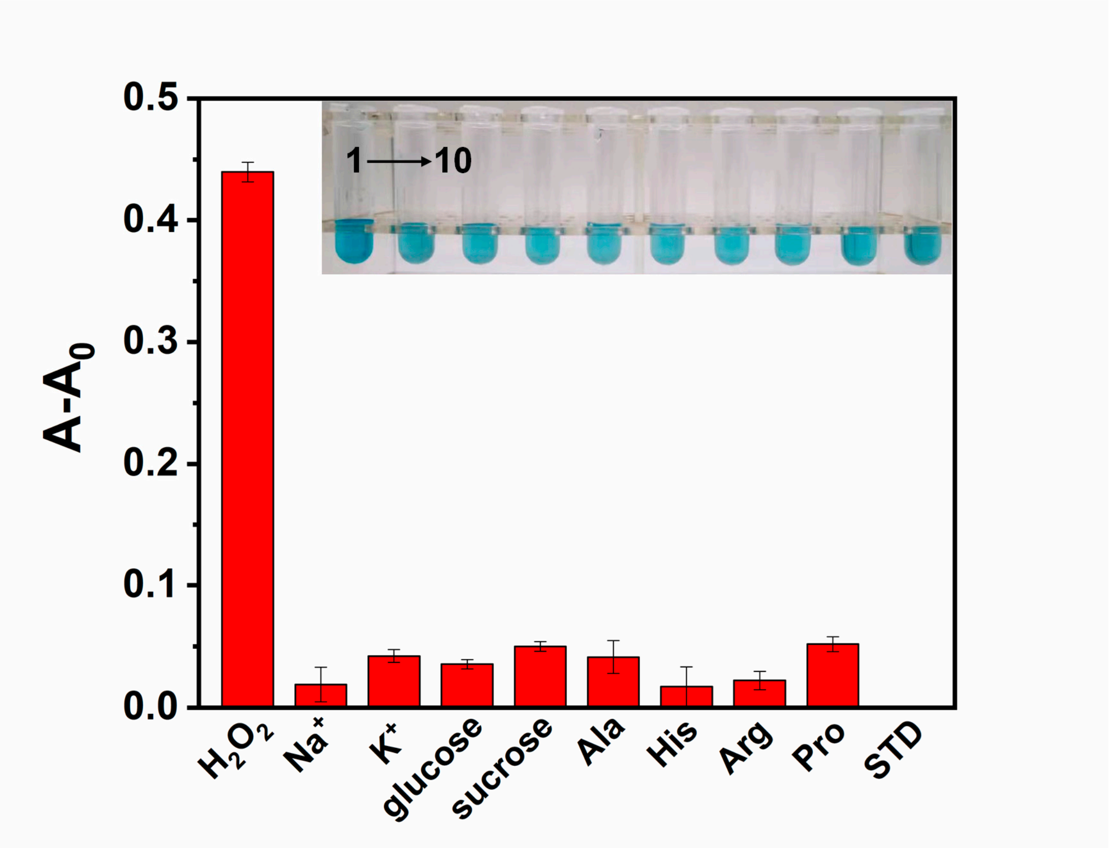

Fig. S13 Variation of the absorbance at 652 nm showing sensing ability of fabricated nanozyme sensor toward H_2O_2 and various interferences (300 μM). Inset: Optical photographs showing sensing ability of fabricated nanozyme sensor toward various interferences (The numerical labels represent the pollutant types marked from left to right on the abscissa, respectively). Conditions: FeCoNi-MOF (25 μg/mL), TMB (0.5 mM) in acetate buffer (pH 4.0), kept at 40 °C for 40 min to reach equilibrium.

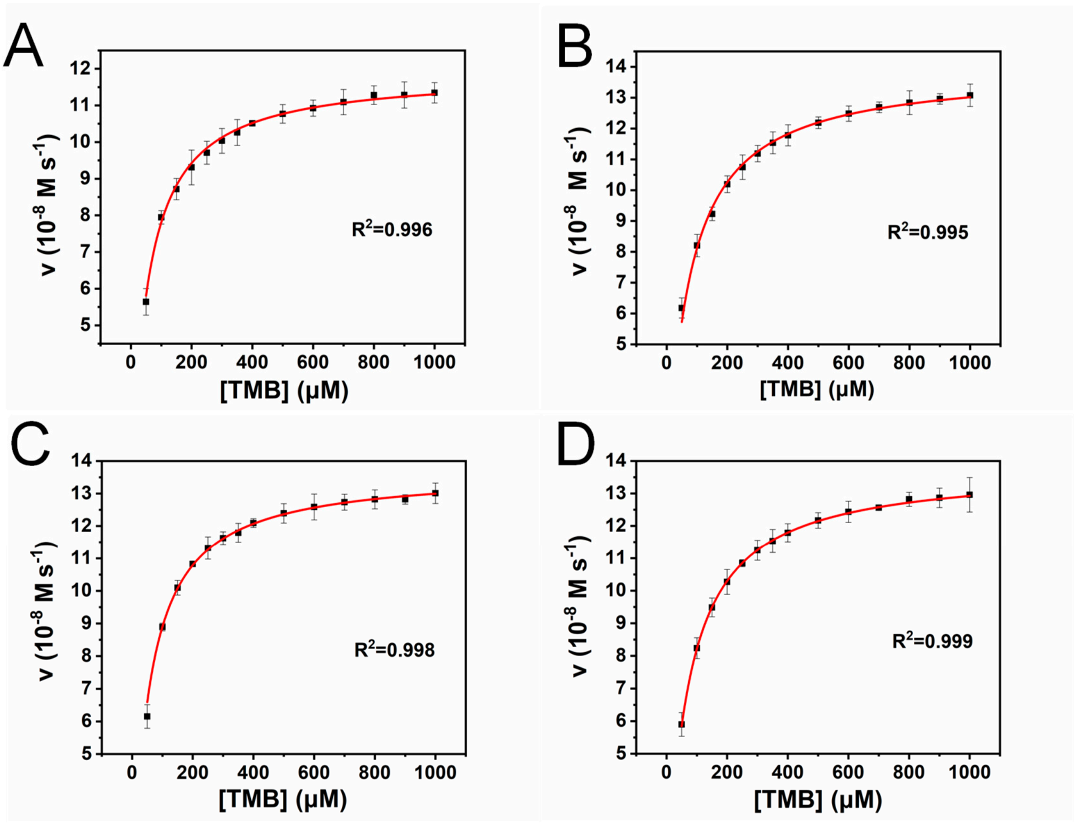

Fig. S14 Steady-state kinetic assay of FeCoNi-MOF as the nanozyme with H_2O_2 and TMB as the substrate: Michaelis-Menten curves of FeCoNi-MOF with the concentration of H_2O_2 fixed and the concentration of TMB varied. Fix the concentration of H_2O_2 at (A) 50 μM; (B) 100 μM; (C) 200 μM; (D) 500 μM.

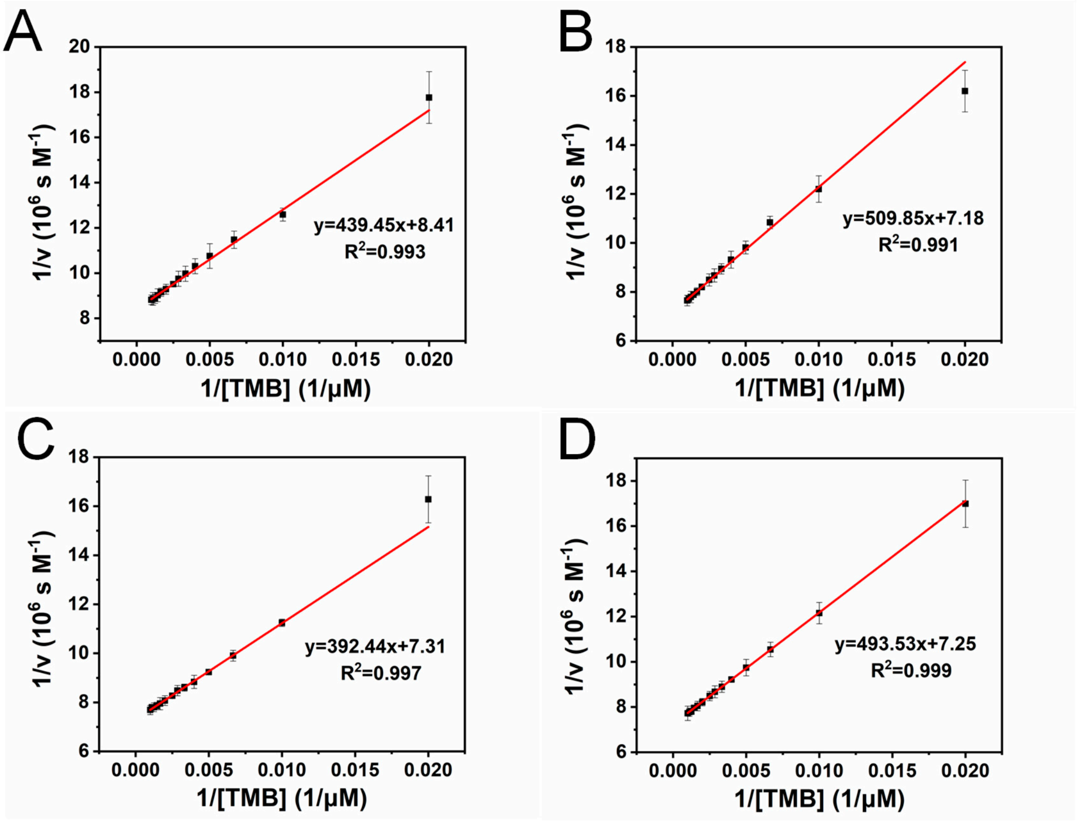

Fig. S15 Steady-state kinetic assay of FeCoNi-MOF as the nanozyme with H_2O_2 and TMB as the substrate: Double-reciprocal plots of FeCoNi-MOF with the concentration of H_2O_2 fixed and the concentration of TMB varied. Fix the concentration of H_2O_2 at (A) 50 μM; (B) 100 μM; (C) 200 μM; (D) 500 μM.

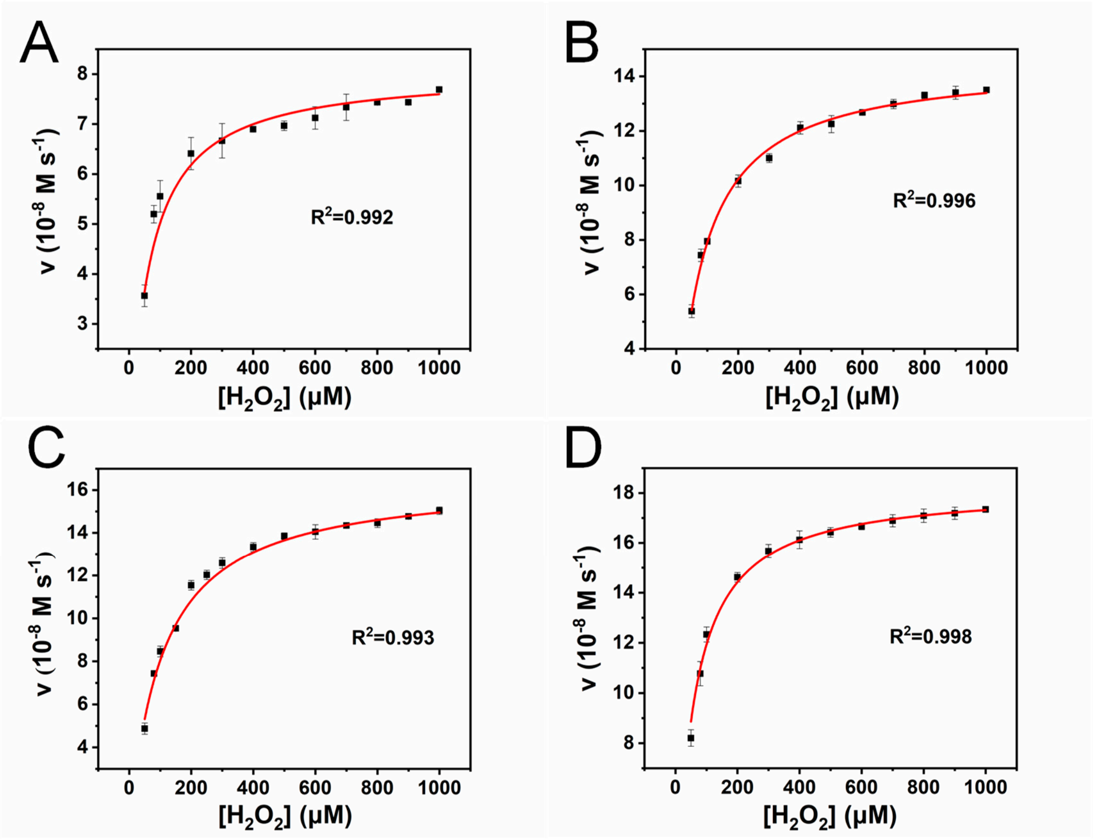

Fig. S16 Steady-state kinetic assay of FeCoNi-MOF as the nanozyme with H_2O_2 and TMB as the substrate: Michaelis-Menten curves of FeCoNi-MOF with the concentration of TMB fixed and the concentration of H_2O_2 varied. Fix the concentration of TMB at (A) 100 μM; (B) 200 μM; (C) 500 μM; (D) 1000 μM.

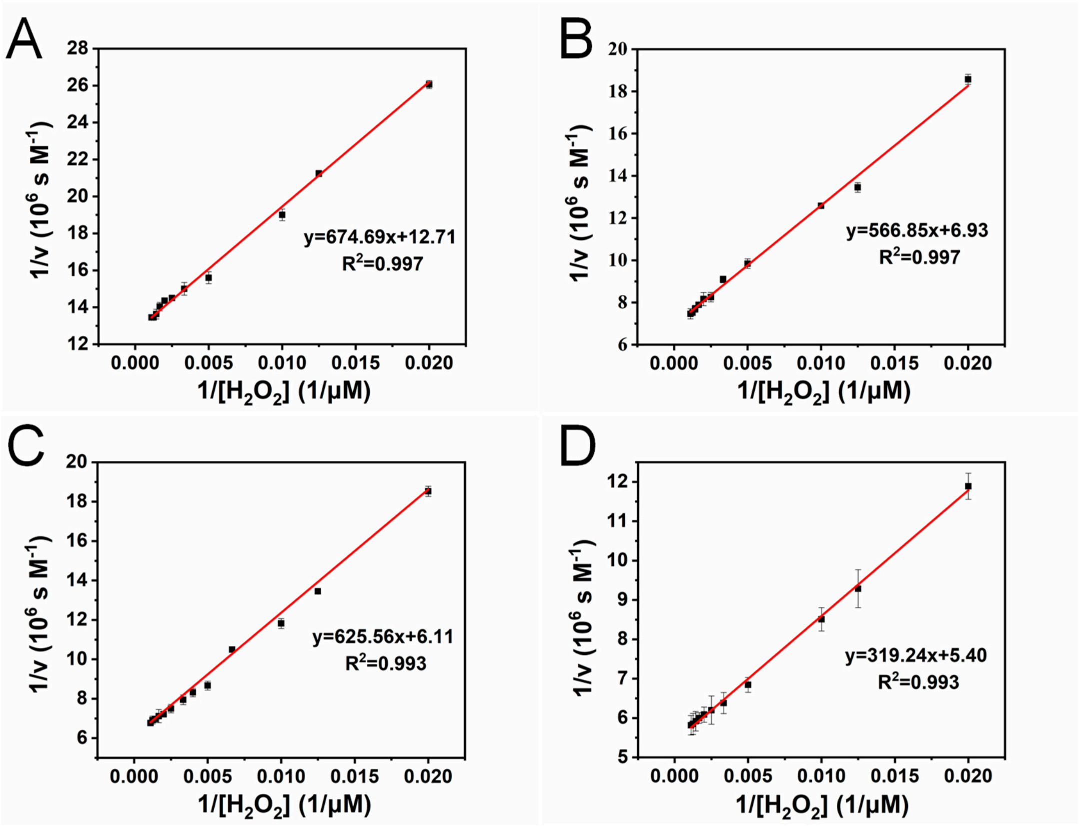

Fig. S17 Steady-state kinetic assay of FeCoNi-MOF as the nanozyme with H_2O_2 and TMB as the substrate: Double-reciprocal plots of FeCoNi-MOF with the concentration of TMB fixed and the concentration of H_2O_2 varied. Fix the concentration of TMB at (A) 100 μM; (B) 200 μM; (C) 500 μM; (D) 1000 μM.

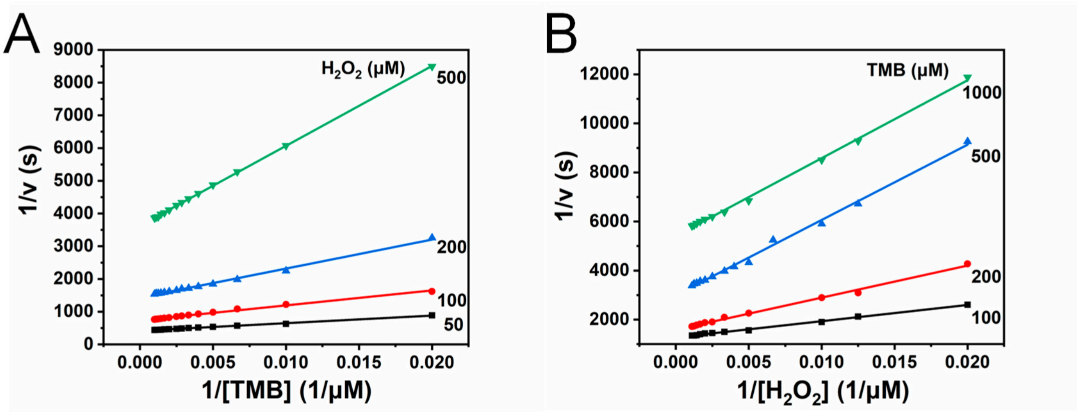

Fig. S18 Double-reciprocal plots of activity of FeCoNi-MOF at a fixed concentration of one substrate versus varying concentration of the second substrate for H_2O_2 and TMB. (A) Fix the concentration of H_2O_2 and vary the concentration of TMB; and (B) fix the concentration of TMB and vary the concentration of H_2O_2.

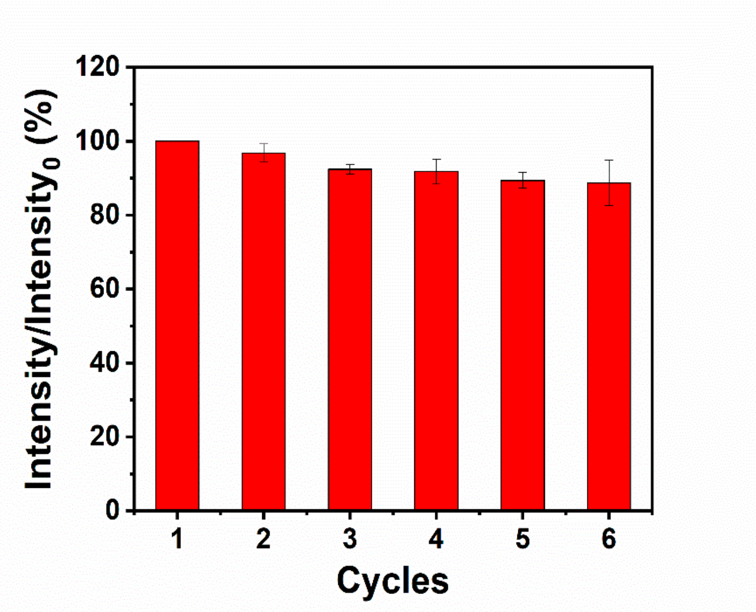

Fig. S19 The catalytic performance of FeCoNi MOF after reuse. Reaction condition: 2 mg mL^{-1} FeCoNi MOF, 500 μM TMB, 500 μM H_2O_2, pH 4.0, 20 min, under 40 °C in 5 mL reaction system.

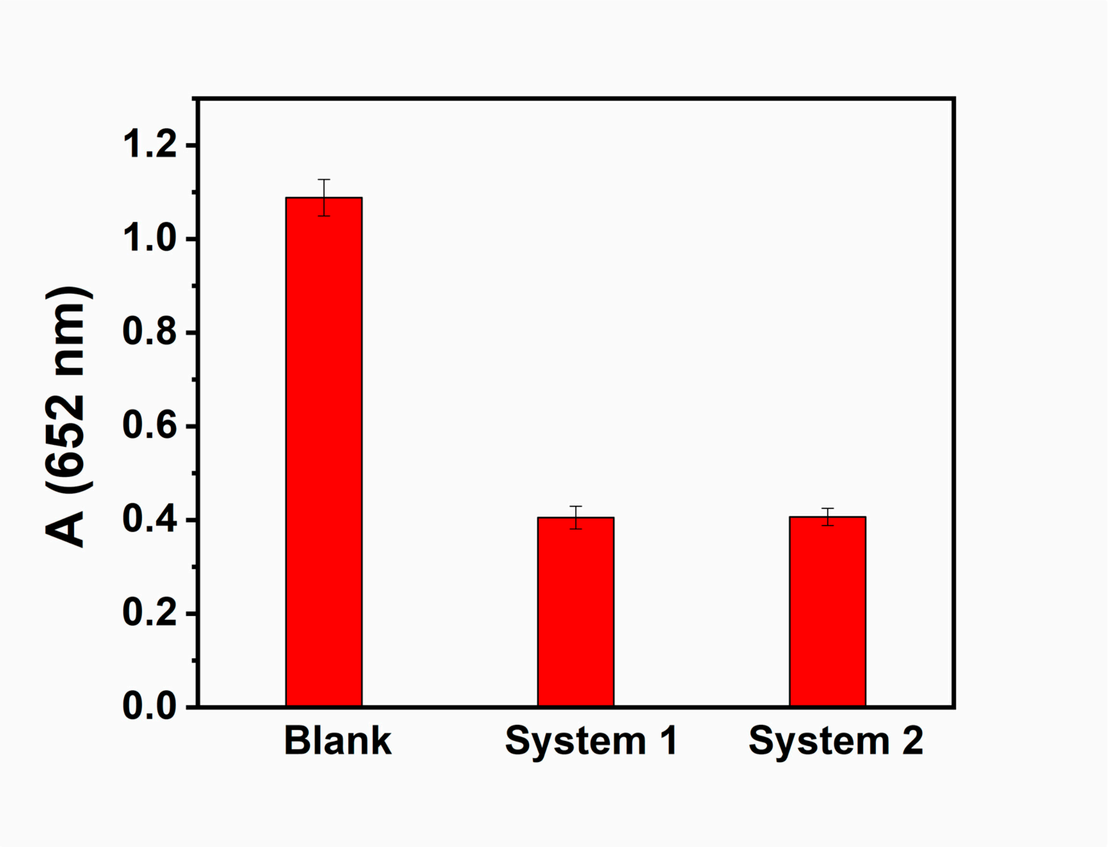

Fig. S20 Absorbance of different systems at 652 nm (suspension of FeCoNi-MOF 25 μg mL^{-1}, TP 50 μM, TMB 500 μM and H_2O_2 400 μM in pH 4.0 acetate buffer (0.1M), the total volume 1 mL). Blank: Without added TP. System 1: TP was added firstly and then TMB, H_2O_2 and suspension of FeCoNi-MOF were added in sequence. Next, the system was incubated at 40 °C for 20 min. System 2: TMB, H_2O_2 and suspension of FeCoNi-MOF were added in sequence and the system was incubated at 40 °C for 20 min similarly. Finally, TP was added and the system continued to incubate at 40 °C for 20 min.

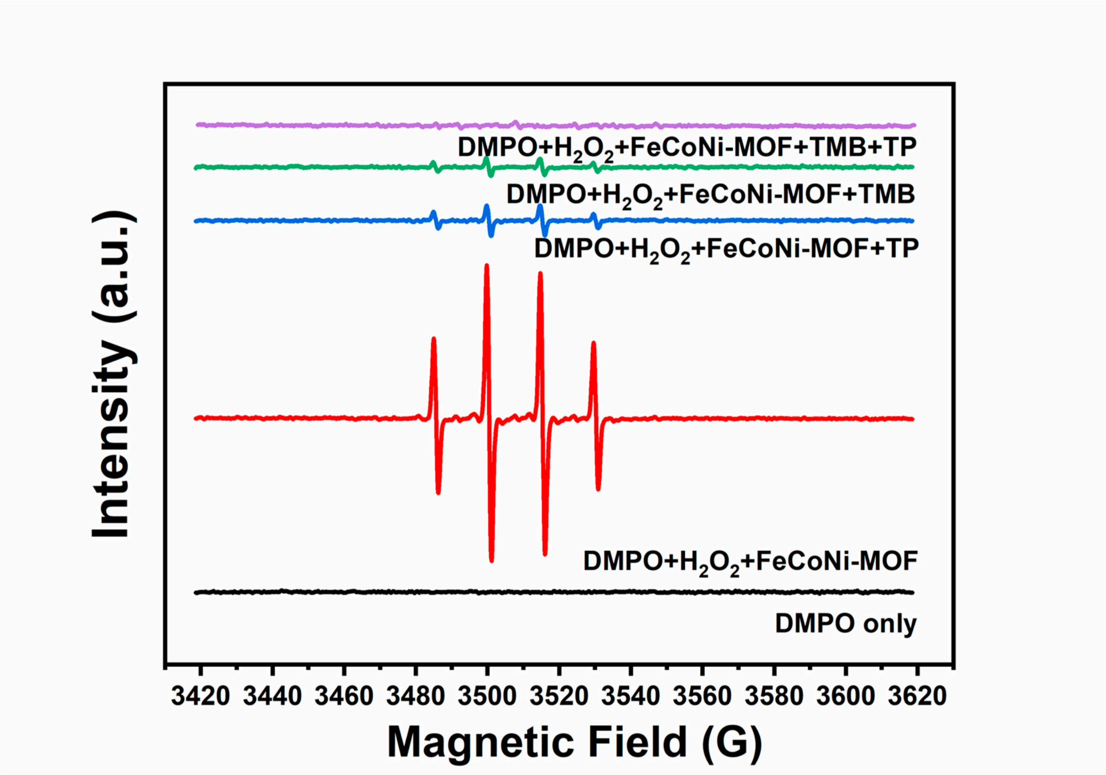

Fig. S21 EPR spectra in the activation process of H_2O_2 under different conditions.

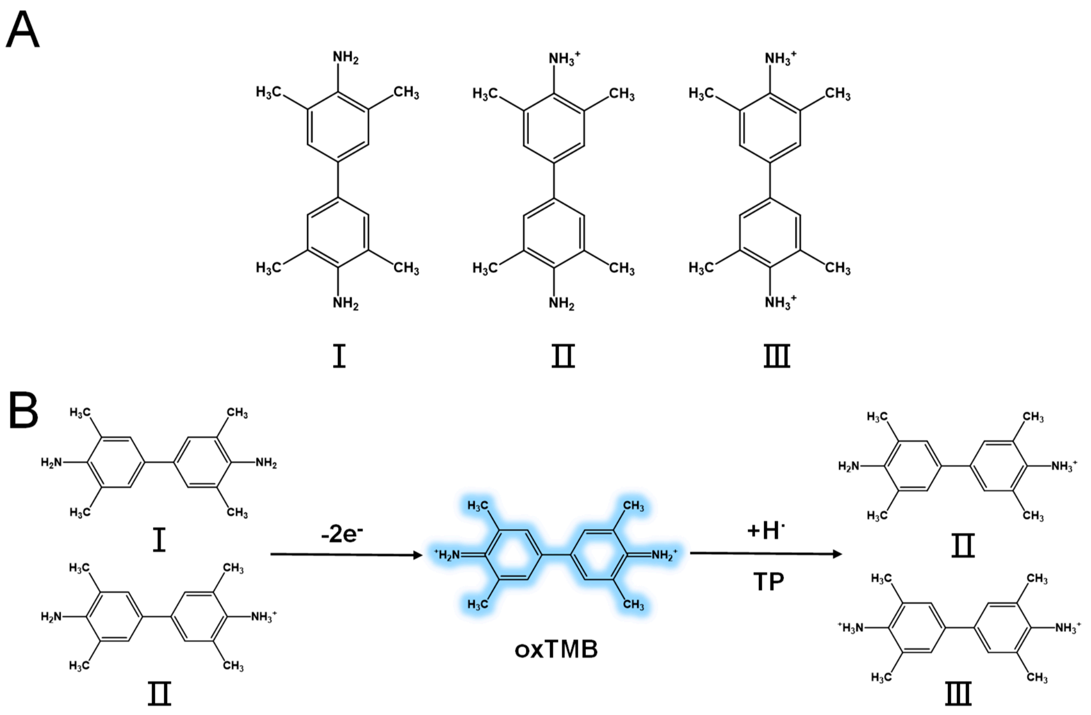

Fig. S22 (A) Three different forms of TMB in acidic pH and (B) mechanism of the oxidation of TMB to oxTMB by free radicals and the restoration of oxTMB to TMB by the hydrogen donation of TP.

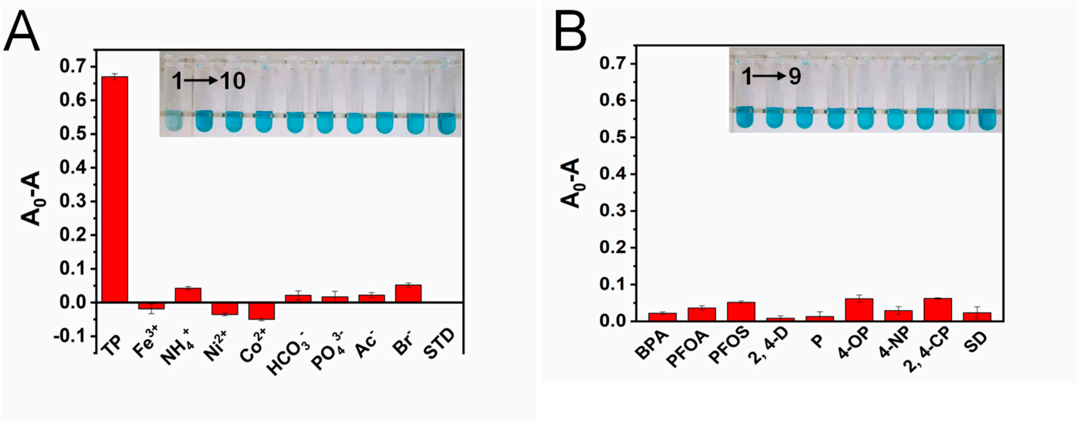

Fig. S23 Variation of the absorbance at 652 nm showing sensing ability of fabricated nanozyme-like sensors toward TP and various interferences (50 μM): (A) Common anions and cations; and (B) Common organic pollutants. Inset: Optical photographs showing sensing ability of fabricated nanozyme sensor toward various interferences (The numerical labels represent the pollutant types marked from left to right on the abscissa, respectively). Conditions: FeCoNi-MOF (25 μg mL^{-1}), TMB (0.5 mM) and H_2O_2 (0.4 mM) in acetate buffer (pH 4.0), kept at 40 °C for 40 min to reach equilibrium.

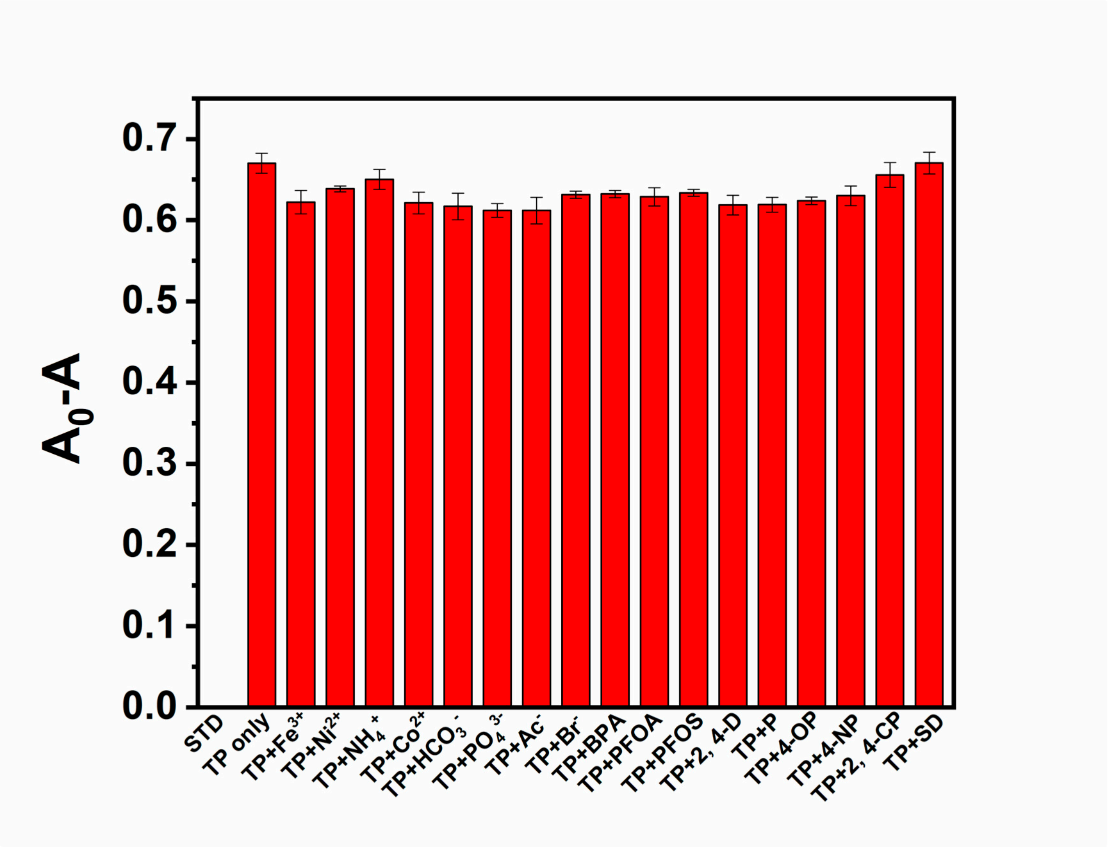

Fig. S24 Variation of the absorbance at 652 nm of the sensing system incubated with TP and various interferences (TP: 50 μM and other interferences: 100 μM). Conditions: FeCoNi-MOF (25 μg mL^{-1}), TMB (0.5 mM) and H_2O_2 (0.4 mM) in acetate buffer (pH 4.0), kept at 40 °C for 40 min to reach equilibrium.

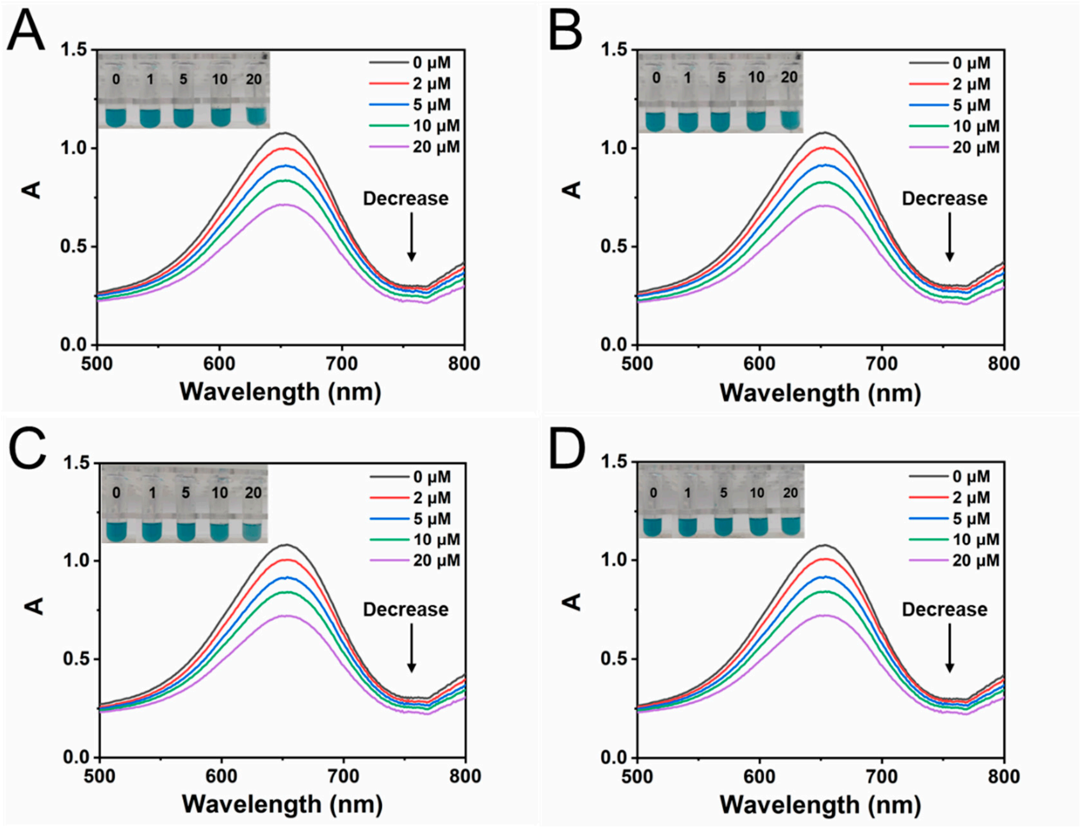

Fig. S25 The UV absorption spectra of different reaction systems of actual water samples spiked with different concentrations of TP (0 µM, 2 µM, 5 µM, 10 µM and 20 µM): (A) Tap water; (B) Jiuxiang River Water; (C) Xuanwu Lake water; (D) Tai Lake water. Inset: Corresponding optical photographs of different reaction systems of actual water samples. Conditions: FeCoNi-MOF (25 µg mL^{-1}), TMB (0.5 mM), H_2O_2 (0.4 mM) in acetate buffer (pH 4.0), kept at 40 °C for 40 min to reach equilibrium.

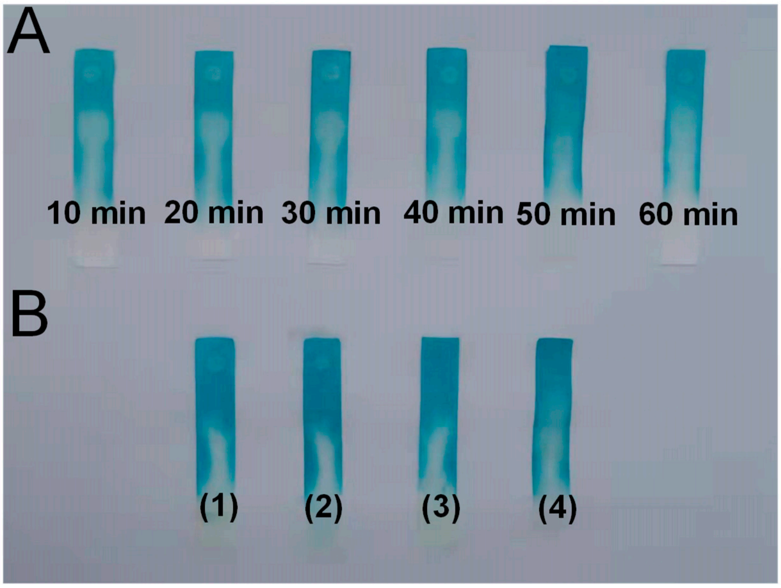

Fig. S26 (A) Activity of paper trip sensor at varying fabrication time (min) (using 2 μL, TP conc., 0.5 mM); (B) Activity of paper sensor in presence of mixture of other pollutants (Fe^{3+}, NH_4^+, Ni^{2+}, HCO_3^-, PO_4^{3-}, Ac^-, PFOA, P, 2, 4-D, 2, 4-CP, 0.5 mM): (1) 2 μL of pure TP solution (0.5 mM); (2) 2 μL of the mixture of TP and other pollutants (containing TP and each pollutant of 0.5 mM). (3) 2 μL of the mixture of other pollutants (containing each pollutant of 0.5 mM); (4) Standard solution without TP and other pollutants.

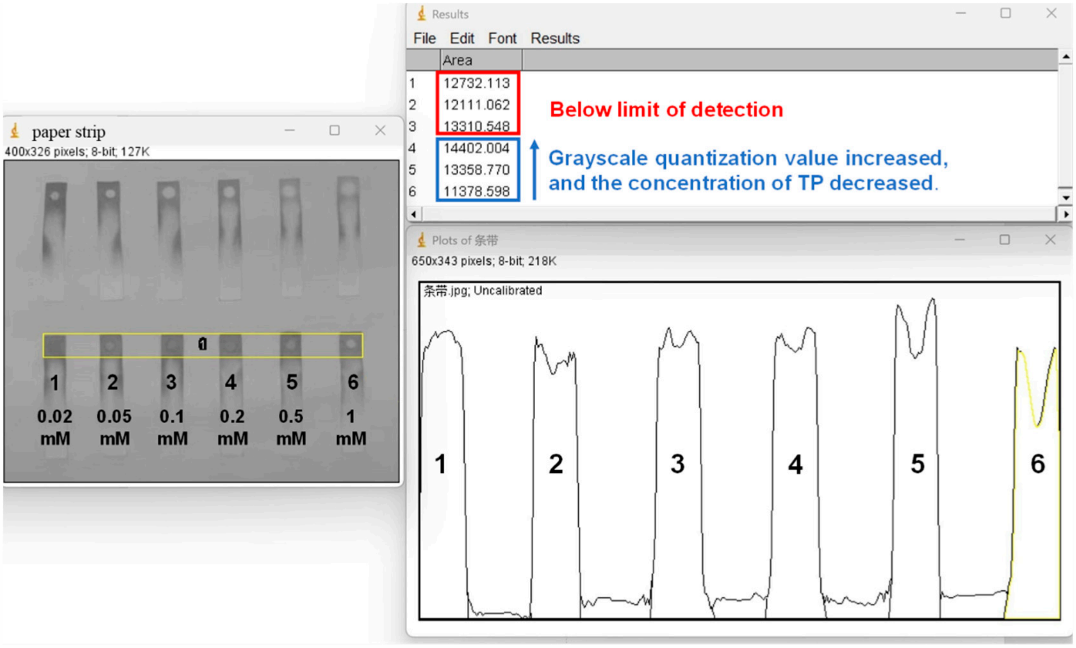

Fig. S27 Analysis of the paper strip sensor by ImageJ_v1.8.0.

Supplementary Tables

Table S1 The peroxidase-like and oxidase-like activities of seven MOFs

	Oxidase-like activity (Absorbance (652 nm) of systems without H_2O_2)	Absorbance (652 nm) of systems with H_2O_2	Peroxidase-like activity (The difference of absorbance (652 nm) between the systems with and without H_2O_2)
Fe MOF	0.6305	0.8701	0.2396
Co MOF	0.6305	0.8344	0.2039
Ni MOF	0.6538	0.7375	0.0837
FeCo MOF	0.6998	1.0382	0.3384
FeNi MOF	0.6640	0.9933	0.3293
CoNi MOF	0.6357	0.9206	0.2849
FeCoNi MOF	0.6883	1.186	0.4977

Table S2 Method comparisons for the analysis of the H_2O_2

Materials	Analytical Methods	Linear range (μM)	LOD (μM)	Reference
HBF-1-C800[a]	Colorimetry	10-1000	7.7	**[3]**
N-Doped Carbon Nanozymes	Colorimetry	20-200	15	**[7]**
Perylene diimide-functionalized CeO_2 nanocomposite	Colorimetry	20-80	2.59	**[8]**
FePt-Au HNPs[b]	Colorimetry	20-700	12.33	**[9]**
Fe^{3+}-MCNs[c]	Colorimetry	5-200	2.63	**[10]**
GDYO[d]	Colorimetry	20-180	20	**[11]**
Fe-AL[e]	Colorimetry	10000-100000	54	**[12]**
Vitamin B3	Colorimetry	5-100	3	**[13]**
$H_2TCPP-Co_9S_8$[f]	Colorimetry	10-200	8.19	**[14]**
Fe-doped Ag_2S	Colorimetry	10-150	7.82	**[15]**
FeCoNi-MOF	Colorimetry	6-800	1.75	This work

[a] A hydrogen-bonded biohybrid framework (HBF) obtained from confining BSA into well-defined HOFs (hydrogen-bonded organic frameworks);
[b] FePt-Au hybrid nanoparticles;
[c] Fe^{3+}-mesoporous carbon nanospheres;
[d] Graphdiyne oxide;
[e] Fe^{3+}-doped aminated lignin;
[f] Two-dimensional porphyrin-Co_9S_8 nanocomposites.

Table S3 Comparison of K_m values and V_{max} values with TMB as the substrate

Materials	Application	V_{max} (10^{-8} M s^{-1})	K_m (mM)	Reference
HRP	Enzyme linked immunosorbent assays (ELISA)	10.00	0.434	**[4]**
Fe_3O_4	ELISA	0.72	0.295	**[18]**
FeS_2	Detection of H_2O_2 and GSH	69.9	4.88	**[19]**
Fe_3O_4@SiO_2@Au	Detection of glucose	1.43	5.71	**[20]**
CS-$MoSe_2$ NS[a]	Detection of Hg^{2+}	46.85	1.317	**[21]**
H-GNs[b]	Detection of Single-Nucleotide Polymorphism	4.55	5.100	**[22]**
NH_2-MIL-88B(Fe)-Ag	Bacterial elimination	2.02	0.5	**[23]**
Glycine-MIL-53(Fe)	Detection of glucose	2.28	0.11	**[24]**
$CuFe_2O_4$	Detection of H_2O_2 and GSH	2.07	2.26	**[25]**
$ZnFe_2O_4$ MNPs	Detection of glucose	13.31	0.85	**[26]**
GOx@HP-MIL-88B-BA[c]	Detection of glucose	6.8	0.22	**[27]**
Fe SAEs[d]	-	5.88	3.92	**[28]**
$Cu_{1.8}S$ NPs	Detection of GSH	-	1.72	**[29]**
Fe-MIL-88NH_2	Detection of glucose	10.47	0.284	**[30]**
GO	-	3.2	0.26	**[31]**
Pt NPs	-	589	0.71	**[31]**
Fe_3O_4	-	40.2	3.9	**[31]**
PCN 222(Fe)	-	92.2	0.17	**[31]**

FeCoNi-MOF	Detection of H_2O_2 and TP	13.32	0.061	This work

[a] chitosan-functionalized molybdenum(IV) selenide nanosheets;

[b] hemin-graphene hybrid nanosheets;

[c] Boronic Acid-Functionalized Hierarchically Porous MOF;

[d] Fe single atom enzymes.

Table S4 Comparison of K_m values and V_{max} values with H_2O_2 as the substrate

Materials	Application	V_{max} (10^{-8} M s^{-1})	K_m (mM)	Reference
HRP	Enzyme linked immunosorbent assays (ELISA)	8.71	3.7	**[4]**
Fe_3O_4	Detection of ATP	12.9	6.66	**[32]**
FeS_2	Detection of H_2O_2 and GSH	39.2	0.227	**[19]**
Fe_3O_4@SiO_2@Au	Detection of glucose	60.88	2.05	**[20]**
CS-$MoSe_2$ NS[a]	Detection of Hg^{2+}	23.26	12.89	**[21]**
H-GNs[b]	Detection of Single-Nucleotide Polymorphism	5.06	2.256	**[22]**
NH_2-MIL-88B(Fe)-Ag	Bacterial elimination	1.77	0.738	**[23]**
Glycine-MIL-53(Fe)	Detection of glucose	2.25	0.1	**[24]**
$CuFe_2O_4$	Detection of H_2O_2 and GSH	2.61	0.5	**[25]**
$ZnFe_2O_4$ MNPs	Detection of glucose	7.74	1.66	**[26]**
GOx@HP-MIL-88B-BA[c]	Detection of glucose	7.2	0.38	**[27]**
Fe SAEs[d]	-	8.25	0.243	**[28]**
$Cu_{1.8}S$ NPs	Detection of GSH	-	37.1	**[29]**
Fe-MIL-88NH_2	Detection of glucose	7.04	0.206	**[30]**
$Pt_{0.1}$/Au NPs	Detection of Hg^{2+}	0.0664	0.436	**[33]**
GO	-	3.2	2.76	**[31]**
Pt NPs	-	602	596.6	**[31]**
Fe_3O_4	-	41	1.0	**[31]**

PCN-222(Fe)	-	92.2	0.17	**[31]**
FeCoNi-MOF	Detection of H_2O_2 and TP	14.30	0.074	This work

[a] chitosan-functionalized molybdenum(IV) selenide nanosheets;

[b] hemin-graphene hybrid nanosheets;

[c] Boronic Acid-Functionalized Hierarchically Porous MOF;

[d] Fe single atom enzymes.

Table S5 Method comparisons for the analysis of the TP

Materials	Analytical Methods	Linear range (μM)	LOD (μM)	Reference
Water-soluble fluorescent probe[a]	Fluorometry	1-50	0.071	**[34]**
Near-infrared xanthene-based fluorescent probe	Fluorometry	2-18	0.120	**[35]**
Coelenterate luciferin-based luminescent probe	Fluorometry	0.5-20	0.65	**[36]**
Triarylborane (TAB) based probe 1	Fluorometry	1-13	0.08	**[37]**
Triarylborane (TAB) based probe 2	Fluorometry	1-15	0.15	**[37]**
Probe YY[b]	Fluorometry	0-160	0.84	**[38]**
Near-infrared (NIR) fluorescent probes QN-DNP	Fluorometry	5-30	0.078	**[39]**
SN-DNP probe[c]	Colorimetry	0.0-7.0	0.55	**[40]**
CN-DNP probe[d]	Colorimetry	0-10	0.12	**[41]**
**1oo** probe[e]	Fluorometry	0-30	2.43	**[42]**
FeCoNi-MOF	Colorimetry	0.5-80	0.045	This work

[a] 1,8-naphthylimide moiety modified with carboxyl to obtain a water-soluble fluorescent probe;
[b] Probe YY was synthesized through a three-step method, which consists of the α,β-unsaturated ethanoylcoumarin fluorophore and a strong electron-withdrawing 2,4-dinitrobenzene group;
[c] A novel spiropyran-based colorimetric probe SP-DNP bearing a 2,4-dinitrophenyl group;
[d] A new fluorescence resonance energy transfer (FRET)-based ratiometric fluorescent probe;
[e] A kind of luminescent probe based on photochromic cyclometalated iridium (III) complex.

Table S6 Analytical data of the sensor

Item		Absorbance			RSD (%)
		1	2	3	
Reproducibility	Intra day	1.186	1.072	1.166	6.09
	Inter day	1.172	1.210	1.106	5.26
Repeatability		1.186	1.119	1.143	3.39

Table S7 Properties of the distilled water and actual water samples

Samples	pH	Salinity (PSU)	EC[a] (μS/cm)	TDS[b] (mg/L)	DO[c] (mg/L)	TOC[d] (mg/L)
Distilled water	6.56	0.01	0.79	0.41	8.63	0.562
Tap water	7.58	0.16	341	172.4	7.62	5.13
Xuanwu Lake	7.88	0.21	485	225	3.98	8.162
Tai Lake	8.14	0.27	528	276	4.15	8.437
Jiuxiang River	8.29	0.29	615	323	4.79	10.934

[a] Electrical conductivity;

[b] Total dissolved solids;

[c] Dissolved oxygen;

[d] Total organic carbon.

Table S8 The grayscale quantization values of three paper strip sensors

Concentration	Paper strip 1	Paper strip 2	Paper strip 3	Mean value	Standard deviation
10 mM	5762.548	5385.390	4988.370	5378.769	316.091
8 mM	6621.719	6124.924	5837.271	6194.638	324.021
5 mM	7002.012	7481.267	6891.741	7125.007	255.904
4 mM	8407.841	8819.238	8012.156	8413.078	329.510
3 mM	9743.134	9230.569	8923.653	9299.119	338.044
2 mM	10749.598	10457.459	9876.124	10361.060	363.050
1 mM	10914.669	11378.598	10782.646	11025.304	255.564
0.5 mM	12629.861	13358.770	11965.382	12651.338	569.050
0.2 mM	13670.690	14402.004	13819.234	13963.976	315.613
0.1 mM	12185.912	13310.548	11781.389	12425.950	646.939
0.05 mM	12164.416	12111.062	10892.451	11722.643	587.438
0.02 mM	11693.426	11732.113	11179.892	11535.144	251.696

Note that the photo quality, software processing selection including size and range of selected points, integral area of the selected points, etc., may all affect the signal of the paper strip sensor by ImageJ_v1.8.0 software. Thus, the accuracy of a paper strip sensor and software recognition should further be improved.

References

1. Senthil Raja, D.; Huang, C.-L.; Chen, Y.-A.; Choi, Y.; Lu, S.-Y., Composition-balanced trimetallic MOFs as ultra-efficient electrocatalysts for oxygen evolution reaction at high current densities. *Applied Catalysis B: Environmental* **2020,** 279, 119375.
2. Mesbah, A.; Malaman, B.; Mazet, T.; Sibille, R.; François, M., Location of metallic elements in (Co1−xFex)2(OH)2(C8H4O4): use of MAD, neutron diffraction and 57Fe Mössbauer spectroscopy. *CrystEngComm* **2010,** 12, (10), 3126.
3. Ye, N.; Huang, S.; Yang, H.; Wu, T.; Tong, L.; Zhu, F.; Chen, G.; Ouyang, G., Hydrogen-Bonded Biohybrid Framework-Derived Highly Specific Nanozymes for Biomarker Sensing. *Anal Chem* **2021,** 93, (41), 13981-13989.
4. Gao, L.; Zhuang, J.; Nie, L.; Zhang, J.; Zhang, Y.; Gu, N.; Wang, T.; Feng, J.; Yang, D.; Perrett, S.; Yan, X., Intrinsic peroxidase-like activity of ferromagnetic nanoparticles. *Nat Nanotechnol* **2007,** 2, (9), 577-83.
5. Chen, J.; Shu, Y.; Li, H.; Xu, Q.; Hu, X., Nickel metal-organic framework 2D nanosheets with enhanced peroxidase nanozyme activity for colorimetric detection of H2O2. *Talanta* **2018,** 189, 254-261.
6. Singh, S.; Mitra, K.; Shukla, A.; Singh, R.; Gundampati, R. K.; Misra, N.; Maiti, P.; Ray, B., Brominated Graphene as Mimetic Peroxidase for Sulfide Ion Recognition. *Anal Chem* **2017,** 89, (1), 783-791.
7. Lou, Z.; Zhao, S.; Wang, Q.; Wei, H., N-Doped Carbon As Peroxidase-Like Nanozymes for Total Antioxidant Capacity Assay. *Anal Chem* **2019,** 91, (23), 15267-15274.
8. Lian, J.; Liu, P.; Jin, C.; Shi, Z.; Luo, X.; Liu, Q., Perylene diimide-functionalized CeO2 nanocomposite as a peroxidase mimic for colorimetric determination of hydrogen peroxide and glutathione. *Mikrochim Acta* **2019,** 186, (6), 332.
9. Ding, Y.; Yang, B.; Liu, H.; Liu, Z.; Zhang, X.; Zheng, X.; Liu, Q., FePt-Au ternary metallic nanoparticles with the enhanced peroxidase-like activity for ultrafast colorimetric detection of H2O2. *Sensors and Actuators B: Chemical* **2018,** 259, 775-783.
10. Sang, Y.; Huang, Y.; Li, W.; Ren, J.; Qu, X., Bioinspired Design of Fe3+-Doped Mesoporous Carbon Nanospheres for Enhanced Nanozyme Activity. *Chemistry A European Journal* **2018,** 24, 7259-7263.
11. Ma, W.; Xue, Y.; Guo, S.; Jiang, Y.; Wu, F.; Yu, P.; Mao, L., Graphdiyne oxide: a new carbon nanozyme. *Chem Commun (Camb)* **2020,** 56, (38), 5115-5118.
12. Li, L.; Liu, X.; Zhu, R.; Wang, B.; Yang, J.; Xu, F.; Ramaswamy, S.; Zhang, X., Fe3+-Doped Aminated Lignin as Peroxidase-Mimicking Nanozymes for Rapid and Durable Colorimetric Detection of H2O2. *ACS Sustainable Chemistry & Engineering* **2021,** 9, (38), 12833-12843.
13. Zhang, C.-Y.; Zhang, W.-Y.; Chen, G.-Y.; Chai, T.-Q.; Zhang, H.; Xu, Y.; Yang, F.-Q., Vitamin B3 as a high acid-alkali tolerant peroxidase mimic for

colorimetric detection of hydrogen peroxide and glutathione. *Arabian Journal of Chemistry* **2022**, 103823.
14. Gao, Y.; Jin, C.; Li, X.; Wu, K.; Gao, L.; Lyu, X.; Zhang, X.; Zhang, X.; Luo, X.; Liu, Q., Two-dimensional porphyrin-Co9S8 nanocomposites with synergistic peroxidase-like catalysis: Synthesis and application toward colorimetric biosensing of H2O2 and glutathione. *Colloids and Surfaces A: Physicochemical and Engineering Aspects* **2019,** 568, 248-258.
15. Ding, Y.; Liu, H.; Gao, L.-N.; Fu, M.; Luo, X.; Zhang, X.; Liu, Q.; Zeng, R.-C., Fe-doped Ag2S with excellent peroxidase-like activity for colorimetric determination of H2O2. *Journal of Alloys and Compounds* **2019,** 785, 1189-1197.
16. Shamsipur, M.; Safavi, A.; Mohammadpour, Z., Indirect colorimetric detection of glutathione based on its radical restoration ability using carbon nanodots as nanozymes. *Sensors and Actuators B: Chemical* **2014,** 199, 463-469.
17. Huang, Z.-M.; Cai, Q.-Y.; Ding, D.-C.; Ge, J.; Hu, Y.-L.; Yang, J.; Zhang, L.; Li, Z.-H., A facile label-free colorimetric method for highly sensitive glutathione detection by using manganese dioxide nanosheets. *Sensors and Actuators B: Chemical* **2017,** 242, 355-361.
18. Zhang, Z.; Zhang, X.; Liu, B.; Liu, J., Molecular Imprinting on Inorganic Nanozymes for Hundred-fold Enzyme Specificity. *J Am Chem Soc* **2017,** 139, (15), 5412-5419.
19. Huang, X.; Xia, F.; Nan, Z., Fabrication of FeS2/SiO2 Double Mesoporous Hollow Spheres as an Artificial Peroxidase and Rapid Determination of H2O2 and Glutathione. *ACS Appl Mater Interfaces* **2020,** 12, (41), 46539-46548.
20. Luo, S.; Liu, Y.; Rao, H.; Wang, Y.; Wang, X., Fluorescence and magnetic nanocomposite Fe3O4@SiO2@Au MNPs as peroxidase mimetics for glucose detection. *Anal Biochem* **2017,** 538, 26-33.
21. Huang, L.; Zhu, Q.; Zhu, J.; Luo, L.; Pu, S.; Zhang, W.; Zhu, W.; Sun, J.; Wang, J., Portable Colorimetric Detection of Mercury(II) Based on a Non-Noble Metal Nanozyme with Tunable Activity. *Inorg Chem* **2019,** 58, (2), 1638-1646.
22. Guo, Y.; Deng, L.; Li, J.; Guo, S.; Wang, E.; Dong, S., Hemin-Graphene Hybrid Nanosheets with Intrinsic Peroxidase-like Activity for Label-free Colorimetric Detection of Single-Nucleotide Polymorphism. *ACS Nano* **2011,** 5, (2), 1282-1290.
23. Zhang, W.; Ren, X.; Shi, S.; Li, M.; Liu, L.; Han, X.; Zhu, W.; Yue, T.; Sun, J.; Wang, J., Ionic silver-infused peroxidase-like metal-organic frameworks as versatile "antibiotic" for enhanced bacterial elimination. *Nanoscale* **2020,** 12, (30), 16330-16338.
24. Dong, W.; Yang, L.; Huang, Y., Glycine post-synthetic modification of MIL-53(Fe) metal-organic framework with enhanced and stable peroxidase-like activity for sensitive glucose biosensing. *Talanta* **2017,** 167, 359-366.
25. Xia, F.; Shi, Q.; Nan, Z., Facile synthesis of Cu-CuFe2O4 nanozymes for sensitive assay of H2O2 and GSH. *Dalton Trans* **2020,** 49, (36), 12780-12792.
26. Su, L.; Feng, J.; Zhou, X.; Ren, C.; Li, H.; Chen, X., Colorimetric detection of

urine glucose based ZnFe2O4 magnetic nanoparticles. *Anal Chem* **2012,** 84, (13), 5753-8.

27. Zhao, Z.; Huang, Y.; Liu, W.; Ye, F.; Zhao, S., Immobilized Glucose Oxidase on Boronic Acid-Functionalized Hierarchically Porous MOF as an Integrated Nanozyme for One-Step Glucose Detection. *ACS Sustainable Chemistry & Engineering* **2020,** 8, (11), 4481-4488.
28. Zhao, C.; Xiong, C.; Liu, X.; Qiao, M.; Li, Z.; Yuan, T.; Wang, J.; Qu, Y.; Wang, X.; Zhou, F.; Xu, Q.; Wang, S.; Chen, M.; Wang, W.; Li, Y.; Yao, T.; Wu, Y.; Li, Y., Unraveling the enzyme-like activity of heterogeneous single atom catalyst. *Chem Commun (Camb)* **2019,** 55, (16), 2285-2288.
29. Zou, H.; Yang, T.; Lan, J.; Huang, C., Use of the peroxidase mimetic activity of erythrocyte-like Cu1.8S nanoparticles in the colorimetric determination of glutathione. *Analytical Methods* **2017,** 9, (5), 841-846.
30. Liu, Y. L.; Zhao, X. J.; Yang, X. X.; Li, Y. F., A nanosized metal-organic framework of Fe-MIL-88NH(2) as a novel peroxidase mimic used for colorimetric detection of glucose. *Analyst* **2013,** 138, (16), 4526-31.
31. Wang, Y.; Li, T.; Wei, H., Determination of the Maximum Velocity of a Peroxidase-like Nanozyme. *Anal Chem* **2023,** 95, (26), 10105-10109.
32. Li, S.; Zhao, X.; Yu, X.; Wan, Y.; Yin, M.; Zhang, W.; Cao, B.; Wang, H., Fe3O4 Nanozymes with Aptamer-Tuned Catalysis for Selective Colorimetric Analysis of ATP in Blood. *Anal Chem* **2019,** 91, (22), 14737-14742.
33. Chen, H.; Qiu, Q.; Sharif, S.; Ying, S.; Wang, Y.; Ying, Y., Solution-Phase Synthesis of Platinum Nanoparticle-Decorated Metal-Organic Framework Hybrid Nanomaterials as Biomimetic Nanoenzymes for Biosensing Applications. *ACS Appl Mater Interfaces* **2018,** 10, (28), 24108-24115.
34. Yan, H.; Yue, Y.; Yin, C.; Zhang, Y.; Chao, J.; Huo, F., A water-soluble fluorescent probe for the detection of thiophenols in water samples and in cells imaging. *Spectrochim Acta A Mol Biomol Spectrosc* **2020,** 229, 117905.
35. Wu, Y.; Shi, A.; Liu, H.; Li, Y.; Lun, W.; Zeng, H.; Fan, X., A novel near-infrared xanthene-based fluorescent probe for detection of thiophenol in vitro and in vivo. *New Journal of Chemistry* **2020,** 44, (40), 17360-17367.
36. Yuan, M.; Ma, X.; Jiang, T.; Zhang, C.; Chen, H.; Gao, Y.; Yang, X.; Du, L.; Li, M., A novel coelenterate luciferin-based luminescent probe for selective and sensitive detection of thiophenols. *Org Biomol Chem* **2016,** 14, (43), 10267-10274.
37. Pagidi, S.; Kalluvettukuzhy, N. K.; Thilagar, P., Triarylboron Anchored Luminescent Probes: Selective Detection and Imaging of Thiophenols in the Intracellular Environment. *Langmuir* **2018,** 34, (28), 8170-8177.
38. Yang, Y.; Feng, Y.; Qiu, F.; Iqbal, K.; Wang, Y.; Song, X.; Wang, Y.; Zhang, G.; Liu, W., Dual-Site and Dual-Excitation Fluorescent Probe That Can Be Tuned for Discriminative Detection of Cysteine, Homocystein, and Thiophenols. *Anal Chem* **2018,** 90, (23), 14048-14055.
39. Yang, Q. Q.; Ji, N.; Zhan, Y.; Tian, Q. Q.; Cai, Z. D.; Lu, X. L.; He, W., Rational design of a new near-infrared fluorophore and apply to the detection and

imaging study of cysteine and thiophenol. *Anal Chim Acta* **2021,** 1186, 339116.
40. Yang, L.; Li, Y.; Song, H.; Zhang, H.; Yang, N.; Peng, Q.; Ji, L.; He, G., A highly sensitive probe based on spiropyran for colorimetric and fluorescent detection of thiophenol in aqueous media. *Dyes and Pigments* **2020,** 175, 108154.
41. Zhai, Q.; Yang, S.; Fang, Y.; Zhang, H.; Feng, G., A new ratiometric fluorescent probe for the detection of thiophenols. *RSC Advances* **2015,** 5, (114), 94216-94221.
42. Guo, Y.; Zhang, D.; Wang, J.; Lu, H.; Pu, S., Luminescent probe based on photochromic cyclometalated iridium(III) complex for high selectivity detection of thiophenol. *Dyes and Pigments* **2020,** 175, 108191.
